# Spiral volumetric optoacoustic tomography of reduced oxygen saturation in the spinal cord of M83 mouse model of Parkinson’s disease

**DOI:** 10.1007/s00259-024-06938-w

**Published:** 2024-10-09

**Authors:** Benjamin F. Combes, Sandeep Kumar Kalva, Pierre-Louis Benveniste, Agathe Tournant, Man Hoi Law, Joshua Newton, Maik Krüger, Rebecca Z. Weber, Inês Dias, Daniela Noain, Xose Luis Dean-Ben, Uwe Konietzko, Christian R. Baumann, Per-Göran Gillberg, Christoph Hock, Roger M. Nitsch, Julien Cohen-Adad, Daniel Razansky, Ruiqing Ni

**Affiliations:** 1 Institute for Regenerative Medicine, University of Zurich, Zurich, Switzerland; 2 Institute for Biomedical Engineering, University of Zurich & ETH Zurich, Zurich, Switzerland; 3 Institute of Pharmacology and Toxicology, University of Zurich, Zurich, Switzerland; 4 NeuroPoly Lab, Institute of Biomedical Engineering, Polytechnique Montreal, Montreal, QC Canada; 5 Mila - Quebec AI Institute, Montreal, QC Canada; 6 Department of Neurology, University Hospital Zurich, University of Zurich, Zurich, Switzerland; 7 Neuroscience Center Zurich (ZNZ), University of Zurich, Zurich, Switzerland; 8 Center of Competence Sleep and Health Zurich, University of Zurich, Zurich, Switzerland; 9 Department of Neurobiology, Care Sciences and Society, Karolinska Institute, Stockholm, Sweden; 10 Neurimmune, Schlieren, Switzerland; 11 Department of Nuclear Medicine, Inselspital, Bern University Hospital, University of Bern, Bern, Switzerland

**Keywords:** Alpha-synuclein, Deep learning, Magnetic resonance imaging, Optoacoustic imaging, Oxygen saturation, Parkinson’s disease, Spinal cord

## Abstract

**Purpose:**

Metabolism and bioenergetics in the central nervous system play important roles in the pathophysiology of Parkinson’s disease (PD). Here, we employed a multimodal imaging approach to assess oxygenation changes in the spinal cord of the transgenic M83 murine model of PD overexpressing the mutated A53T alpha-synuclein form in comparison with non-transgenic littermates.

**Methods:**

In vivo spiral volumetric optoacoustic tomography (SVOT) was performed to assess oxygen saturation (sO_2_) in the spinal cords of M83 mice and non-transgenic littermates. Ex vivo high-field T1-weighted (T1w) magnetic resonance imaging (MRI) at 9.4T was used to assess volumetric alterations in the spinal cord. 3D SVOT analysis and deep learning-based automatic segmentation of T1w MRI data for the mouse spinal cord were developed for quantification. Immunostaining for phosphorylated alpha-synuclein (pS129 α-syn), as well as vascular organization (CD31 and GLUT1), was performed after MRI scan.

**Results:**

In vivo SVOT imaging revealed a lower sO_2_^SVOT^ in the spinal cord of M83 mice compared to non-transgenic littermates at sub-100 μm spatial resolution. Ex vivo MRI-assisted by in-house developed deep learning-based automatic segmentation (validated by manual analysis) revealed no volumetric atrophy in the spinal cord of M83 mice compared to non-transgenic littermates at 50 μm spatial resolution. The vascular network was not impaired in the spinal cord of M83 mice in the presence of pS129 α-syn accumulation.

**Conclusion:**

We developed tools for deep-learning-based analysis for the segmentation of mouse spinal cord structural MRI data, and volumetric analysis of sO_2_^SVOT^ data. We demonstrated non-invasive high-resolution imaging of reduced sO_2_^SVOT^ in the absence of volumetric structural changes in the spinal cord of PD M83 mouse model.

**Supplementary Information:**

The online version contains supplementary material available at 10.1007/s00259-024-06938-w.

## Introduction

Parkinson’s disease (PD) is the second most common neurodegenerative disease affecting 1 to 3% of the elderly population (≥ 60 years old), with the prevalence increasing over the past generation [1]. PD is clinically characterized by bradykinesia, rigidity, and resting tremor [1], which is pathologically characterized by the accumulation of Lewy bodies composed of insoluble alpha-synuclein (α-syn) fibrils. Emerging evidence shows a close relationship between a reduction in the oxygen supply, α-syn pathology, and PD development [2, 3]. Oxygen intake and utilization disorders, such as cerebral hypoperfusion [4] and hypoxia in the brain, have been implicated in the pathogenesis of PD [2, 5]. Risk genes for PD, such as leucine-rich-repeat kinase 2, *PINK1*, and *PRKN* [6], are often associated with mitochondrial dysfunction and impaired cellular respiration. In addition, environmental risk factors associated with PD, including air pollution, pesticide, and heavy metal exposure, are directly linked to oxygen uptake and utilization disorders by modulating ventilation, competing for haemoglobin binding, or affecting the mitochondrial electron transport chain [7–9]. Cerebrovascular risk factors such as prior stroke, hypertension, coronary heart disease, or congestive heart failure are also associated with PD, and targeting them is currently considered a promising approach to slow pathology [10, 11]. According to the Braak staging of PD based on the spreading of alpha-synuclein (α-syn) pathology, the spinal cord is affected early in the disease process (stage 2) prior to the nigrostriatal pathway (stage 3) and limbic system (stage 4) in the brain [12, 13]. This is also associated with functional connectivity changes [14–16], which may contribute to clinical non-motor and motor symptoms commonly developing in PD patients, including pain, constipation, and poor balance [17], indicating its importance in disease progression.

Recent in vitro studies have shown that hypoxic stress-induced leads to abnormal accumulation of the phosphorylated alpha-synuclein at the position Ser129 (pS129 α-syn) and α-syn oligomers, as well as degeneration of dopaminergic neurons [18, 19]. Transient focal ischemia has also been shown to upregulate pS129 α-syn in a stroke mouse model [20]. In humans, studies have shown increased plasma levels of total α-syn and pS129 α-syn in patients suffering from chronic intermittent hypoxia or obstructive sleep apnea [21], which are predisposing factors for PD development. Further exploration of hypoxia signaling mechanisms in α-syn pathology and neurodegeneration will facilitate a better understanding of PD pathogenesis and allow for the exploration of new therapeutic strategies.

A number of imaging modalities, such as perfusion and functional magnetic resonance imaging (MRI) [22–24], two-photon phosphorescence lifetime microscopy [25, 26], positron emission tomography [27], contrast-enhanced [28] and ultrasound (US) localization microscopy [29], have previously been employed for imaging functional alterations in the spinal cord of rodent models. Optoacoustic imaging has previously been employed to study hemodynamic alterations in neurodegenerative diseases [30] and stroke [31–33], as well as other proteinopathies using extrinsic contrast agents [34, 35]. This method has also been used to detect changes in the spinal cord of an ischemic stroke mouse model [36, 37], as well as in an experimental autoimmune encephalomyelitis model of multiple sclerosis [38]. Spiral volumetric optoacoustic tomography (SVOT) represents a state-of-the-art approach for whole-body preclinical volumetric 3D imaging with scalable spatio-temporal resolution [39] with nearly isotropic resolution and rich spectroscopic optical contrast, making it highly suitable for accurate signal quantification and direct oxygenation measurements in the mouse body spinal cord.

The current study aims to develop tools to assess potential oxygenation changes and volumetric reduction in the spinal cord of α-synucleinopathy mouse model [39, 40]. We used the M83 PD mouse model (hetero- and homozygous), which overexpresses the mutated A53T α-syn form, 9–12 months of age) [41]. We assessed spinal oxygenation by mapping in vivo with SVOT (at sub-100 μm spatial resolution) [42, 43], and analyzed the sO_2_^SVOT^ using in-house developed processing and volumetric analysis script. This was followed by ex vivo T1-weighted (T1w) MRI (at 50 μm spatial resolution) and in-house developed deep learning-based gray matter (GM) and white matter (WM) segmentation quantification, as well as immunofluorescence staining for validation *ex vivo.*

## Methods

### Animal model

In total, 22 M83 mice (14 homozygous and 8 heterozygous) between 9 and 12 months of age overexpressing the A53T-mutated human alpha-synuclein gene SNCA gene under the mouse prion promoter (C57Bl/C3H background) [37, 41] and 13 age-matched non-transgenic littermates (NTLs) of both sexes (M83 10/12 males/females and NTLs 6/7 males/females) were used throughout the study. Heterozygous and homozygous M83 mice develop motor impairment at 22–28 and 8–16 months of age, respectively [37, 41]. The brain histopathology and development of behavioral abnormalities have been well characterized in the transgenic M83 PD mouse model [41]. Motor symptoms are thought to be an outcome of pyramidal or motor neurodegeneration rather than the result of nigrostriatal degeneration in this model [44] and α-syn inclusions are found primarily in the spinal cord and the brain stem. Four M83 (2/2 males/females) and three NTLs (0/3 males/females) were scanned using the in vivo SVOT system, 15 M83 (9/6) and 7 NTLs (2/5) were imaged ex vivo with MRI, and 9 M83 (3/6 males/females) and 6 NTLs (4/2 males/females) were included in the histological analysis. The animals were housed in groups in individually ventilated cages inside a temperature-controlled room under a 12-h dark/light cycle. Pelleted food (3437PXL15, Cargill) and water were provided *ad libitum*. All the experiments were performed in accordance with the Swiss Federal Act on Animal Protection and were approved by the Cantonal Veterinary Office Zurich (ZH024/2021). In accordance with the animal license and animal housing facility requirements, we monitored the well-being and checked weekly for whether there was presence of paralysis in the mice, as well as before the imaging experiment. None of the animals displayed paralysis of the limbs, which is consistent with previously reported observations in M83 mice at this age [41].

### Spiral volumetric optoacoustic tomography

The SVOT system employs a Nd: YAG-pumped optical parametric oscillator laser (SpitLight, Innolas Laser GmbH, Krailling, Germany) as an excitation light source. It delivers < 10 ns duration pulses at a repetition rate of 10 Hz over a broad tunable wavelength range (680–1250 nm) with per-pulse energies up to ~ 180 mJ. Five wavelengths (700, 730, 760, 800 and 850 nm) were employed repeatedly in succession (20 averages per wavelength) to efficiently unmix the oxygenated haemoglobin (HbO_2_), deoxygenated haemoglobin (HbR), and total haemoglobin components using the absorption spectra of HbO_2_ and HbR [45]. The light beam was guided through a custom-made fiber bundle (CeramOptec GmBH, Bonn, Germany) placed at a radial distance of 40 mm from the center of a custom-made spherical array. The output bundle created a Gaussian illumination profile with a size of 10 mm at full width at half maximum on the mouse skin surface. The optical fluence on the skin surface was maintained within safe limits according to the American National Standards Institute regulations throughout all experiments [46]. A custom-made spherical array, comprising 512 distinct piezoelectric sensor elements, each with a surface area of ~ 7 mm², a central detection frequency of 7 MHz, and a detection bandwidth of ~ 85% (spanning from 2.6 to 8.6 MHz at the full width at half maximum), was employed to collect the optoacoustic responses [40]. These elements were organized on a hemispherical surface with a radius of 40 mm and an angular coverage of 110 degrees (0.85π solid angle). Simultaneously, the optoacoustic signals were digitized at a rate of 40 Mega samples per second using a tailored parallel data acquisition unit, Falkenstein Mikrosysteme GmBH, Taufkirchen, Germany). This data acquisition unit was synchronized with the laser’s Q-switch output and connected to a PC via a 1 Gb/s Ethernet link for data storage and subsequent analysis. The data acquisition process was performed with a custom MATLAB interface (Version R2020b, MathWorks Inc., Natick, MA, USA) running on a PC. The mice (*n* = 4 M83 and *n* = 3 NTLs) were imaged according to a protocol published earlier [39, 40]. Anesthesia was induced via the use of 4% isoflurane (gas flow rate of 800 ml/min air + 200 ml/min oxygen mix, 3–4 min), with the mouse placed in the induction box with a heating pad beneath. The temperature of the mice was monitored before the scan (within 36 ± 0.5 °C). All the mice were immediately placed under constant inhalation anesthesia with 1.5% isoflurane (inhalation gas flow rate 400 ml/min air + 100 ml/min oxygen) after induction of anesthesia. The whole period including imaging preparation and image acquisition lasted for 50 min for all the mice (under 1.5% isoflurane). Inhalation anesthesia was delivered in a closed system with a thin transparent membrane covering the nose and mouth of the animal, allowing it to breathe normally [33, 39, 40]. The mice were fixed on the animal holder and the preparation took approximately 10 min. The mice were given another 10 min to stabilize before imaging acquisition. The temperature of the mice was monitored during imaging to ensure that the temperature was within the range of 36 ± 0.5 °C throughout the experiments.

### SVOT image reconstruction and spectral unmixing

At each scanning position of the spherical array on the back of the mouse, the signals were initially averaged 20 times for each wavelength (700, 730, 760, 800 and 850 nm), bandpass filtered between 0.1 and 12 MHz, and eventually deconvolved with the impulse response of the spherical array sensing elements. Next, a GPU-implemented backprojection reconstruction technique was employed for the reconstruction of individual volumetric frames, with each US sensing element split into 16 subelements [39, 40]. Whole-spinal cord images were obtained by stitching individual reconstructed volumes at each corresponding position of the spherical array. To quantify the HbO_2_- and HbR levels in the spinal cord, a linear spectral unmixing algorithm was employed [31]. Before unmixing, the reconstructed whole-spinal cord volumes for each wavelength were normalized to the respective optical fluence values.

The oxygen saturation values rendered with SVOT on a voxel-by-voxel basis (sO_2_^SVOT^) were then calculated as (HbO_2_/(HbO_2_ + HbR))*100 and normalized to a 0–1 scale. To quantify the sO_2_^SVOT^ in the spinal cord volumetrically, we further developed code and volumetric analysis pipeline for the reconstruction and processing of the imaging data. In our earlier SVOT studies, the optoacoustic signals were not yet quantified in 3D [39, 40]. All the image reconstruction and processing steps were performed in MATLAB (MathWorks, USA). Maximum intensity projection images (sagittal view) of the grayscale sO_2_^SVOT^ from the MATLAB files were captured, and the mean sO_2_^SVOT^ signal intensities in the spinal cord segments were measured via the atlas of the mouse spinal cord [47] as a reference. Fiji (NIH, USA) was used for visualization.

### Sample Preparation

Immediately following SVOT imaging, M83 and NTLs were intracardially perfused under deep anesthesia with 0.1 M phosphate-buffered saline (PBS, pH 7.4) followed by 4% paraformaldehyde in 0.1 M PBS (pH 7.4). Mouse head and vertebral column samples were postfixed in 4% paraformaldehyde in 0.1 M PBS for 6 days and stored in 0.1 M PBS (pH 7.4) at 4 °C as described earlier [48]. 

### Ex vivo MRI of the M83 mouse head and spinal cord

Mouse head and vertebral column samples were placed in a 15 ml centrifuge tube filled with perfluoropolyether (Fomblin Y, LVAC 16/6, average molecular weight 2,700; Sigma‒Aldrich, USA) [49]. Data were acquired on a BioSpec 94/30 preclinical MRI scanner (Bruker BioSpin AG, Switzerland) with a cryogenic 2 × 2 radio frequency phased-array surface coil (overall coil size of 20 × 27 mm^2^). The coil system operated at 30 K for reception in combination with a circularly polarized 86 mm volume resonator for transmission [50]. For the spinal cord, a structural T1w scan was acquired with a 3D multi-shot echo planar imaging sequence (4 shots) with a field of view = 25 mm × 10 mm × 10 mm and matrix dimension = 500 × 200 × 200, resulting in a nominal voxel resolution of 50 × 50 × 50 μm. The following imaging parameters were chosen: echo time = 8 ms, repetition time = 50 ms, and number of averages = 4. The total acquisition time was 2 h 28 min for each segment [48]. For the brain, a structural T1w scan was acquired with a 3D multishot echo planar imaging sequence (4 shots) with a field-of-view of 18 × 12 × 9 mm and matrix dimension of 180 × 120 × 90, resulting in a nominal voxel resolution of 100 × 100 × 100 μm [51]. 

### MRI data postprocessing and analysis

ITK-SNAP software (v4.0.1, Penn Image Computing and Science Laboratory - PICSL, University of Pennsylvania, Philadelphia, PA) was used to inspect and manually reorient the MR images of the spinal cord. By using an anatomical atlas [47] for guidance, representative axial T1w image sections matching the 23 spinal segments from the thoracic, lumbar and sacral parts (T1-T13, L1-L6 and S1-S4) were identified. GM and WM in the spinal cord were automatically segmented using an in-house developed deep learning-based model trained on manually annotated slices and improved by active learning [52]. The model, which is based on a 3D nnUNet architecture [53], takes as input a 3D MR image and outputs a 3D segmentation file with values of 1 for GM, 2 for WM and 0 for background. The GM and WM cross-sectional areas (CSAs) were then extracted using the sct_process_segmentation command from the Spinal Cord Toolbox [54] (v6.1). Validation of the deep learning method was performed by comparing the automatically computed CSAs (without angle correction) with the manually segmented CSAs from the same 13 thoracic sections (T1-T13) for one mouse. To study the potential volumetric changes, the average of five CSAs from 5 consecutive sections was calculated per segment for all 23 identified segments (thoracic, lumbar and sacral) and per mouse. GM and WM CSAs as well as WM/GM ratios were further analyzed.

### Immunofluorescence and immunohistochemical staining

Mouse brain and spinal cord samples were extracted and placed in 0.1 M PBS for 24 h in 15% sucrose and in 30% sucrose in 0.1 M PBS until the tissue sank or for a maximum of 5 days. Coronal brain Sect. (30 μm) were obtained using a HM 450 Epredia sliding microtome (Thermo Scientific, UK). Spinal cords were embedded in optimal cutting temperature compound in SpineRacks [55] made of natural PVA and printed with the Ultimaker S5 3D printer (Ultimaker B.V.; Geldermalsen, Netherlands). Transverse sectioning (30 μm) was performed using a Leica CM1900 cryostat (Leica Biosystems, Germany). After being cut, free floating sections were stored in 0.1 M PBS + 0.1% sodium azide at 4 °C.

For immunofluorescence staining, the sections were rinsed three times in 0.1 M PBS for 10 min, followed by a 1 h incubation in 0.1 M PBS, 5% v/v normal donkey serum (NDS), 5% v/v normal goat serum (NGS), and 0.5% v/v Triton-X for blocking and permeabilization (information about the antibodies, chemicals and materials used in Supp. Table 1). Primary antibodies against NeuN, glial fibrillary acidic protein (GFAP), cluster of differentiation 31 (CD31), glucose transporter 1 (GLUT1) and pS129 α-syn (EP1536Y, 81A and MJR-R13 clones) were incubated overnight at 4°C (in 0.1 M PBS, 3% v/v NDS, 3% v/v NGS and 0.3% v/v Triton-X) [51, 56]. The sections were then rinsed three times for 10 min each in 0.1 M PBS before being incubated for 2 h in species-specific secondary antibodies (suspended in 0.1 M PBS with 3% v/v NDS, % v/v NGS and 0.3% v/v Triton-X). The tissues were then counterstained with 4’,6-diamidino-2-phenylindole (DAPI) and mounted on microscope slides in Prolong Diamond antifade mounting medium.

For immunochemical staining, the sections were rinsed three times in 0.1 M PBS for 10 min, followed by a 30 min incubation in 0.1 M PBS and 1% H_2_O_2_ before another three washes and a 1 h incubation in 5% v/v NGS and 0.5% v/v Triton-X for blocking and permeabilization. Primary antibodies against Iba1 suspended in 0.1 M PBS, 3% v/v NGS, or 0.3% v/v Triton X-100 were incubated with the samples overnight at 4 °C. The sections were then rinsed three times for 10 min each in 0.1 M PBS before being incubated with a biotinylated anti-mouse secondary antibody (suspended in 0.1 M PBS with 0.5% v/v Triton-X) for 2 h. The sections were then incubated with an avidin-biotin complex–horseradish peroxidase for 1 h at room temperature. The sections were developed with 0.025% 3,3′-diaminobenzidine and 0.05% H_2_O_2_ in triphosphate-buffered saline (TBS, pH 7.4) for 3 min. After being mounted on slides, the sections were dehydrated by an ascending alcohol series of 70%, 90%, and 100% (twice each) and Roticlear^®^ for 2 min. Coverslips were finally mounted with Rotimount^®^ mounting medium.

### Image acquisition and microscopic analysis

For vascular organization analysis, platelet endothelial cell adhesion molecule, also known as CD31 staining and GLUT1 staining of two or three sections per segment (thoracic, lumbar and sacral), were imaged at 10× and 63× magnification using a TCS SP8 confocal laser scanning microscope (Leica, Germany). For CD31 staining, the vascular area fraction, length, and number of branches in the spinal cord section were assessed by using automated analysis as previously described [57]. For GLUT1 staining, the area fraction of the spinal cord section was analyzed. Three or four pictures per segment (thoracic, lumbar and sacral) were taken at 63× magnification using a confocal laser scanning microscope to study the pS129 α-syn-positive signal in the spinal cord sections. Automatic quantification of the colocalization of the pS129-positive pixel with the DAPI-positive pixel was performed with ImageJ (Fiji, NIH) to quantify its presence in the vicinity of the nucleus. To study the relative nuclear fraction of pS129 α-syn, the pS129-positive signal colocalized with DAPI was divided by the DAPI-positive area. This fraction was named the “relative nuclear pS129-positive signal area”. The remaining fraction of the pS129-positive signal was divided by the total area of the image subtracted by the DAPI area to analyze the fraction occupied by this signal in the cytoplasmic compartment (“relative cytoplasmic pS129-positive signal area”). Representative images of CD31, GLUT1 and pS129 α-syn staining of the spinal cord sections were taken using Zeiss Axio Scan Z1 slide scanner (Carl Zeiss Meditec AG, Germany) at 20× magnification and a TCS SP8 confocal laser scanning microscope at 63× magnification. Representative images of GFAP and NeuN staining were taken using TCS SP8 confocal laser scanning microscope at 10× and 20× magnification. Representative images of Iba1 staining were taken using Zeiss Axio Scan Z1 slide scanner at 20× magnification.

### Statistical analysis

All the statistical analyses were performed using GraphPad Prism 9.5.0 (GraphPad Software, Inc., USA). Data distributions were first tested for normality by visually assessing the histograms and the Shapiro‒Wilk test. For distributed data (Figs. 3 and 5), groups were compared using two-way analysis of variance (ANOVA) followed by Holm‒Sidak’s multiple comparisons post hoc analysis. Unpaired Mann‒Whitney tests and Wilcoxon matched-pairs signed rank tests followed by Holm‒Sidak’s multiple comparison *post hoc* analyses were performed for the data shown in Fig. 6B and Supplemental Fig. 4 respectively, as the data were not normally distributed. Nonparametric Spearman’s rank analysis was performed for analyzing the correlation between readouts. Violin plots display all the dots, quartiles and medians, and bar plots present the mean ± standard deviation. Significance was set at *p* < 0.05.

## Results

### Reduction in sO_2_^SVOT^ in the spinal cord of M83 mice

**Fig. 1:**
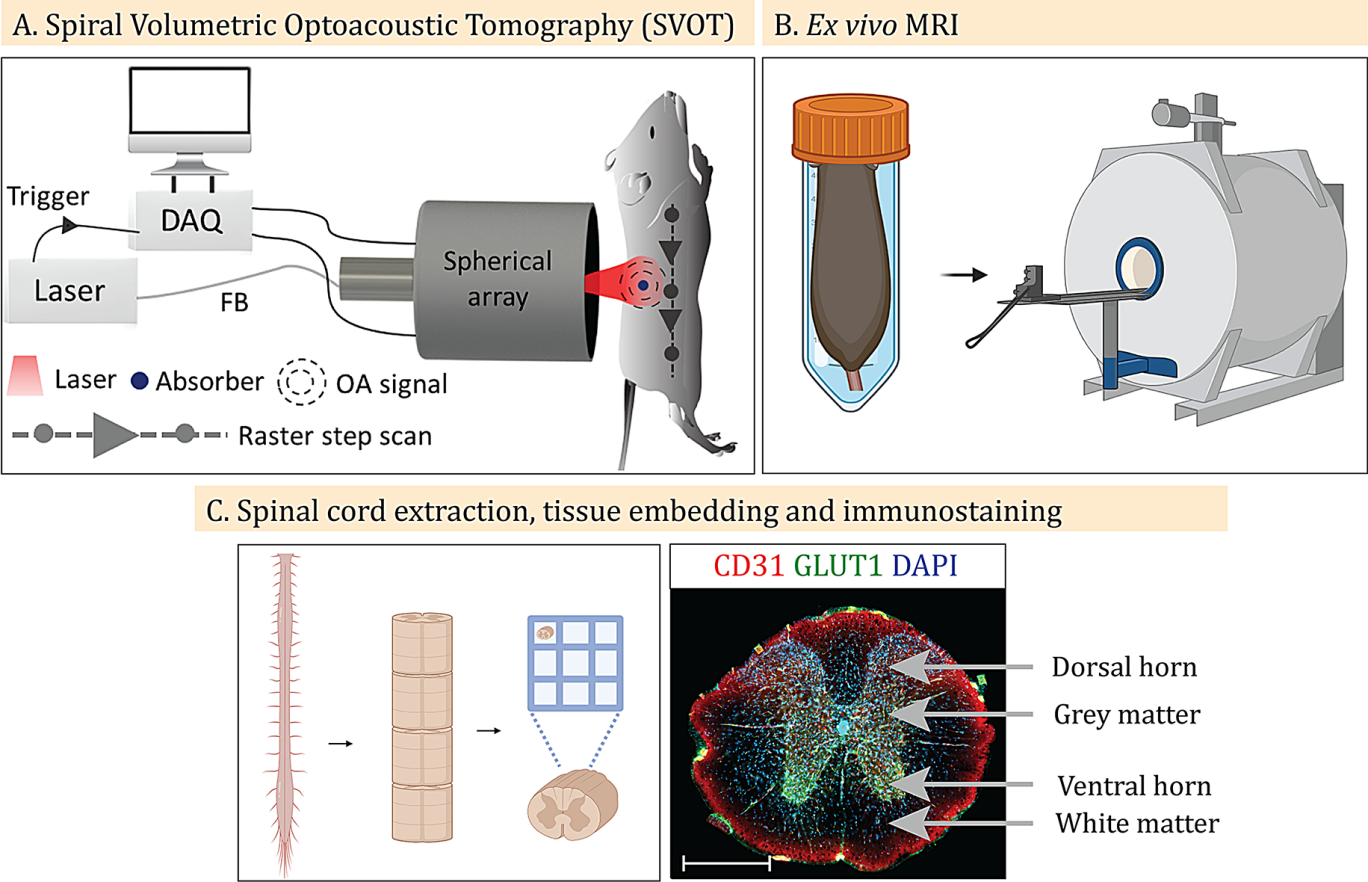
Workflow and experimental setup of the study: **(A)** Schematic of the SVOT system for head-to-tail volumetric imaging of mice. FB: fiber bundle, DAQ: data acquisition system, OA: optoacoustic. **(B)** Ex vivo MRI of the mouse spinal cord. **(C)** Schematic of spinal cord extraction, sectioning, embedding in SpineRacks [55] and immunostaining. Scale bar = 50 μm. (Illustration created on Biorender.com)

**Fig. 2:**
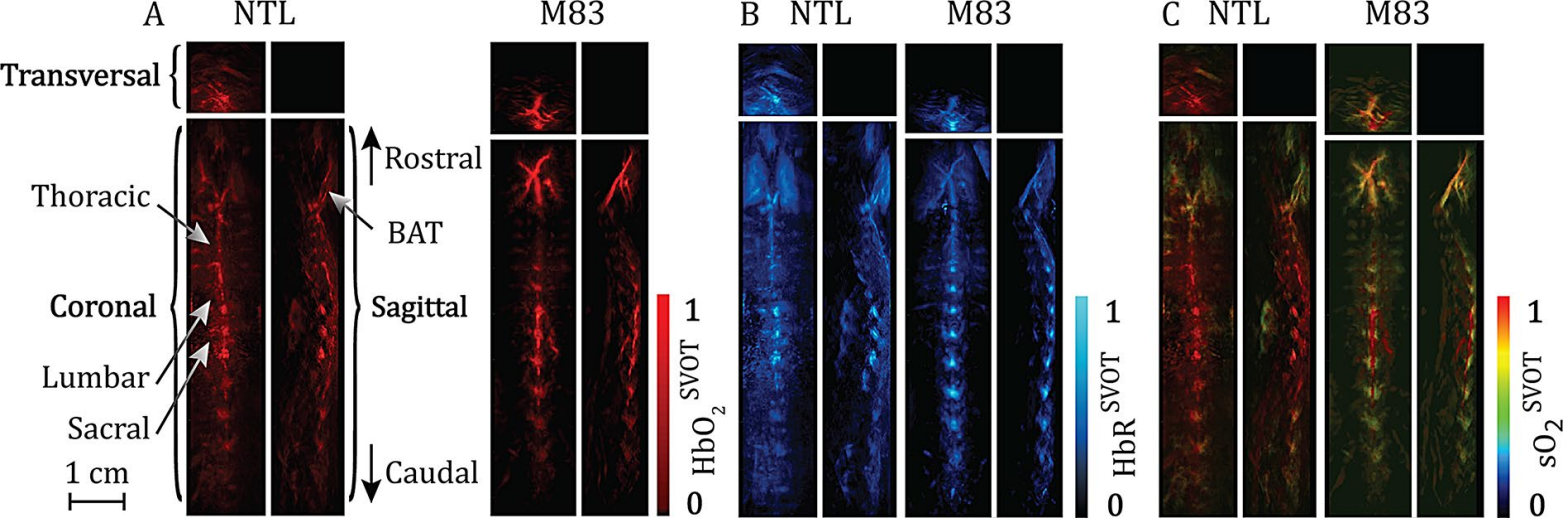
In vivo SVOT imaging of the spinal cords of M83 and NTL (control) mice: **(A-C)** Representative maximum-intensity projection images of HbO_2_**(A)**, HbR **(B)**, and sO_2_^SVOT^**(C)** in the spinal cord of NTL and M83 mice; scale bar = 1 cm. BAT: brown adipose tissue, HbO_2_: oxygenated haemoglobin, HbR: deoxygenated haemoglobin

**Fig. 3:**
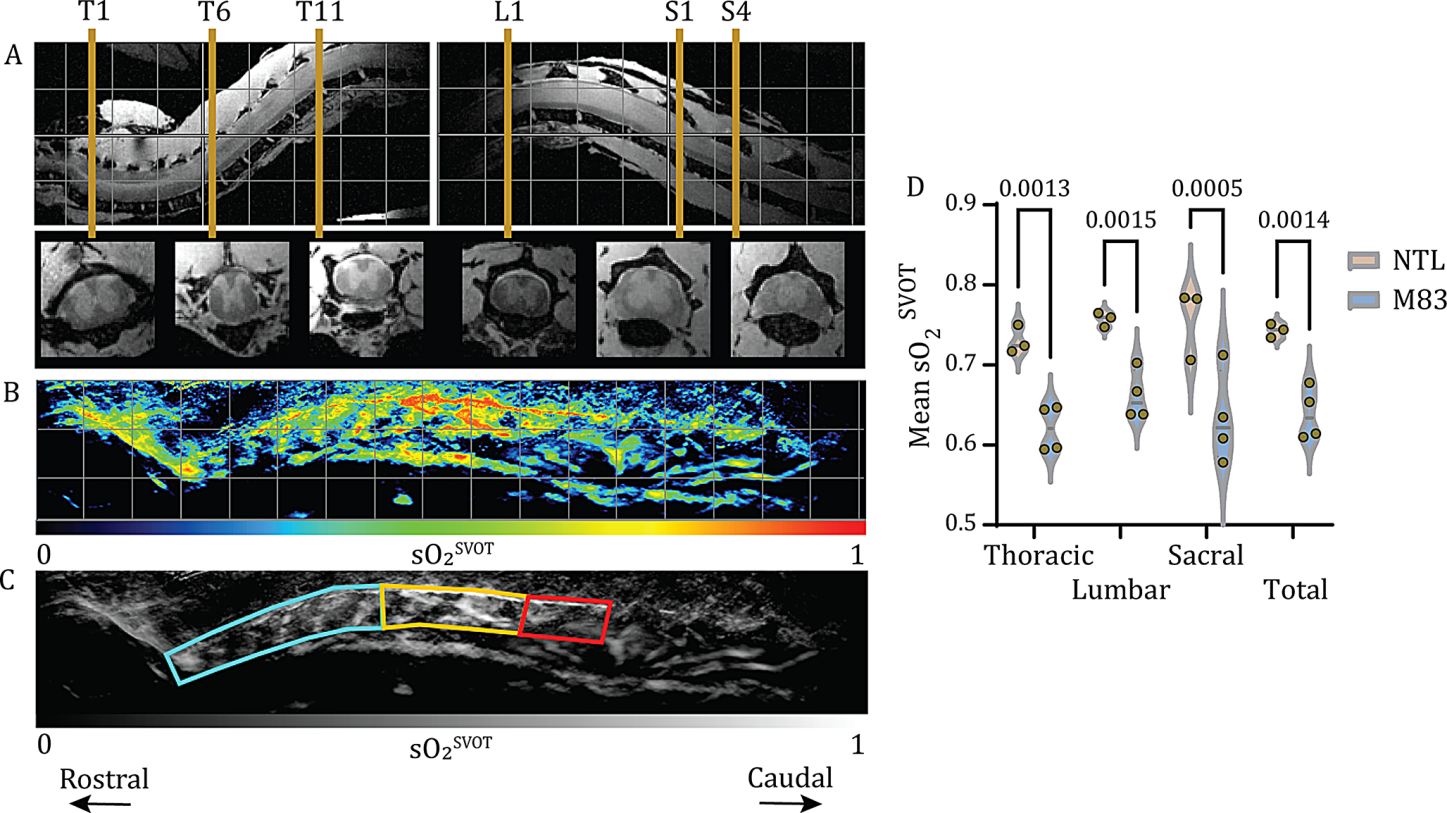
Reduced sO_2_^SVOT^ in the spinal cord of M83 mice compared with NTL (control) mice: **(A)** Ex vivo MR image showing sections of the thoracic (T, left), lumbar (L, right) and sacral (S, right) vertebral segments from a M83 mouse. First row: sagittal view; second row: transverse view; grid = 2.5 mm. The transverse views display six landmarks of the spinal cord (T1, T6, T11, L1, S1 and S4) used for segmentation. **(B)** Representative sO_2_^SVOT^ distribution (sagittal projection) from a M83 mouse; grid = 3 mm. **(C)** Representation of the same images transformed into grayscale with regions-of-interest as markers for further quantification. Cyan: thoracic, orange: lumbar and red: sacral segments. **(D)** Comparison of the mean sO_2_^SVOT^ between M83 and NTL mice in the thoracic, lumbar, and sacral segments and in three combined segments (total). sO_2_^SVOT^ scale: 0–1. *N* = 4 M83 and *n* = 3 NTLs. NTL: non-transgenic littermate

We first assessed in vivo whether alterations in sO_2_^SVOT^ levels occurred in the spinal cord of M83 mice and NTLs at 9–12 months of age (Fig. 1A). SVOT imaging was performed with a spherical matrix array transducer scanned along the animal’s back from neck-to-tail in a step-and-go scanning manner. At each position of the spherical array, multispectral optoacoustic data were spectrally unmixed to differentiate HbO_2_ (Fig. 2A) and HbR (Fig. 2B) contents along the spinal cord [39], followed by calculation of the sO_2_^SVOT^ (Fig. 2C). To visualize the differences in the sO_2_^SVOT^ across the spinal cord segments, ex vivo structural T1w scans were acquired to use landmarks for segmentation (Figs. 1B and 3A). sO_2_^SVOT^ sagittal maximum intensity projections were segmented into thoracic, lumbar, and sacral ROIs (Fig. 3B and C), and the mean sO_2_^SVOT^ was computed (Fig. 3D). The cervical part of the spinal cord could not be assessed as too little optoacoustic signal was measured. This may be due to the brown adipose tissue (BAT) covering this region, which is strongly absorbing based on its high metabolic activity [58], preventing the light from reaching the spinal cord underneath. Compared with age-matched NTL mice, M83 mice presented significant reductions in the sO_2_^SVOT^ in the thoracic (by 15%, *p* = 0.0013), lumbar (by 13%, *p* = 0.0015), and sacral regions (by 16%, *p* = 0.0005) as well as the total spinal cord (by 14%, *p* = 0.0014) (Fig. 3D). These findings suggested a reduction in the sO_2_^SVOT^ level in the spinal cord of M83 mice.

### Low sO_2_^SVOT^ in the spinal cord is not associated with spinal volumetric reduction

**Fig. 4:**
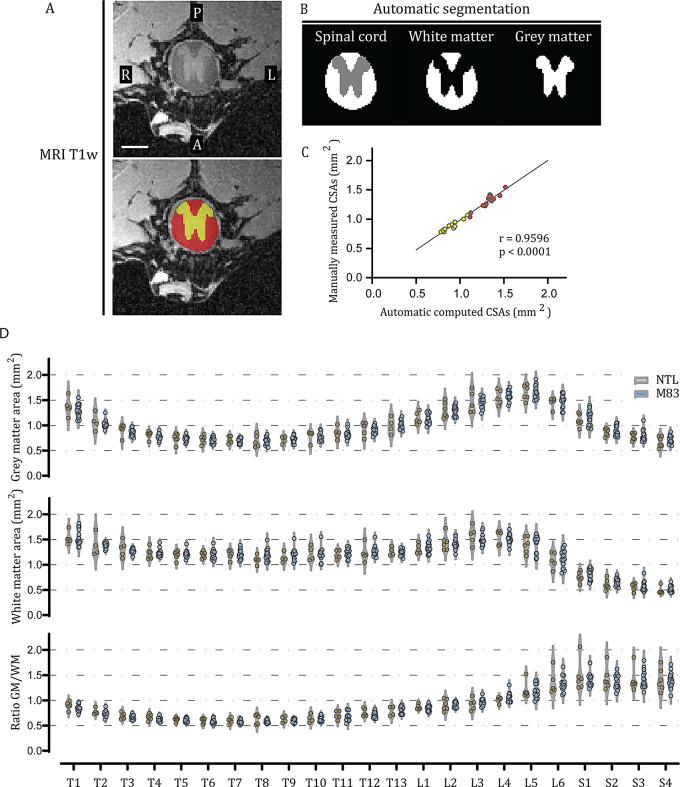
Absence of spinal atrophy in the ex vivo T1w MRI images of M83 mice compared with their NTLs (controls): **(A)** Representative transverse section of the thoracic spinal segment. The upper and lower panels show the same image where manual segmentation is depicted in the lower panel. Yellow: gray matter, red: white matter, R: right, L: left, A: anterior, P: posterior; scale bar = 1 cm. **(B)** Representation of deep learning computed segmentation in the same section shown in A. **(C)** Spearman’s correlation between deep learning-generated and manually segmented CSAs. The yellow and red dots represent values for gray and white matter, respectively. **(D)** Comparison of the mean gray matter cross-sectional areas (upper panel), white matter cross-sectional areas (middle panel) and gray matter/white matter cross-sectional areas (lower panel) between M83 and NTL mice in the thoracic, lumbar, and sacral segments. *N* = 15 M83 and *n* = 7 NTLs. CSA: cross-sectional area, NTL: non-transgenic littermate

Next, we developed an automatic segmentation generated by a deep learning model to quantify the potential spinal volumetric changes associated with low spinal sO_2_^SVOT^ (code and imaging datasets are available). To validate the automatic segmentation generated by the deep learning model, we first compared the CSA values of the GM and WM of the model outputs (Fig. 4B) with those of the manually segmented CSAs (Fig. 4A) in the same sections. Spearman’s correlation analysis revealed a very strong positive linear relationship between the deep learning method and manual segmentation (*n* = 26, *r* = 0.9596, *p* < 0.0001) (Fig. 4C). These findings indicate that the deep learning model, which is used to generate GM and WM CSAs, is an efficient and reliable tool for quantifying potential structural spinal changes in mice.

**Fig. 5:**
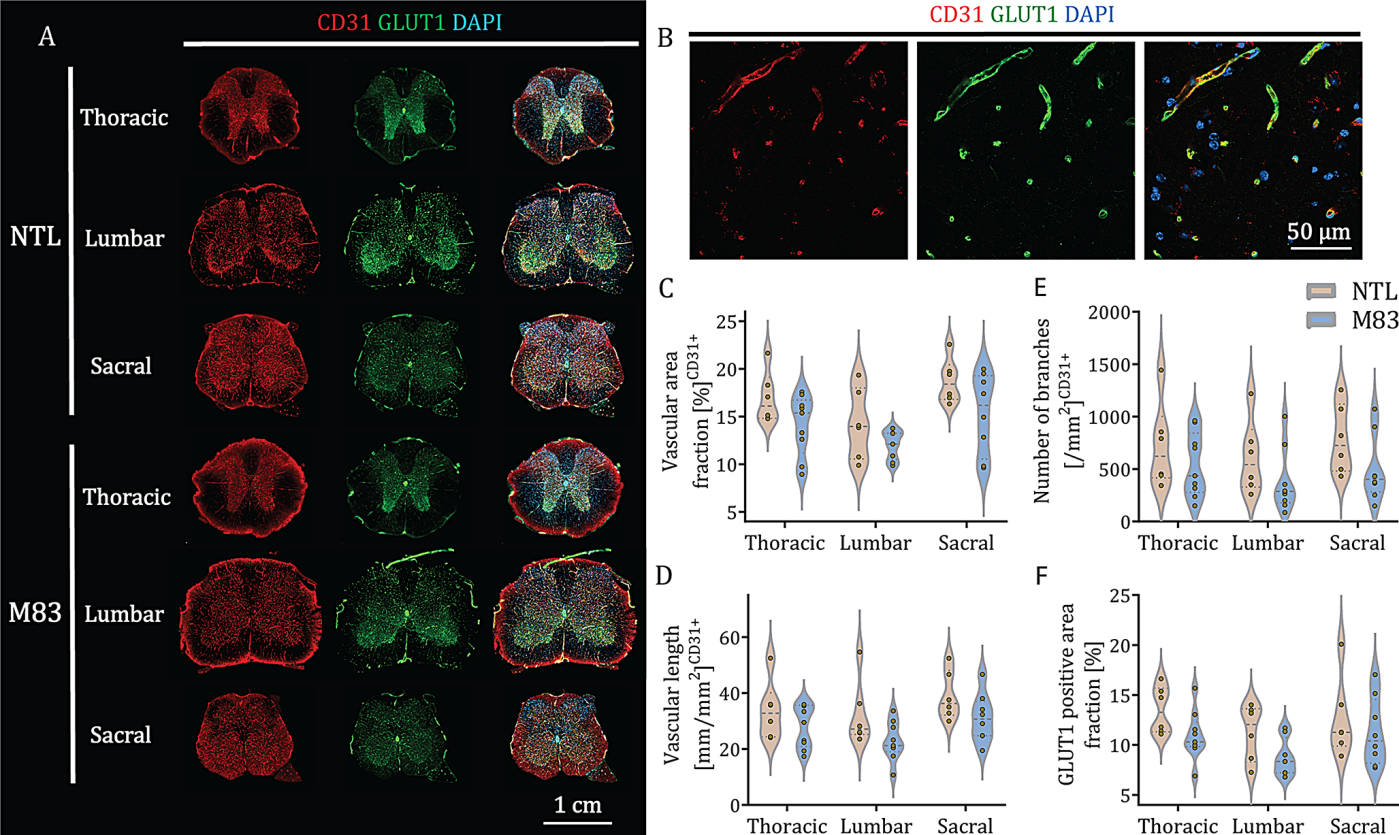
Vascular density, organization and function are not impaired in the spinal cord of M83 mice: **(A)** Representative immunofluorescence images of CD31 (red), GLUT1 (green) and DAPI (blue) at the level of the thoracic, lumbar and sacral vertebrae in NTL and M83 mice with a zoom-in showing close colocalization of CD31 and GLUT1; scale bar = 1 cm. **(B)** Representative immunofluorescence images of CD31 (red), GLUT1 (green) and DAPI (blue) at the level of the thoracic spinal segment in the GM of M83 mice at higher magnification showing close colocalization of CD31 and GLUT1; scale bar = 50 µm. (**C, D** and **E)** Quantitative evaluation of the vascular area fraction, number of branches and blood vessel length in the different spinal segments of both groups. F) Quantitative evaluation of the GLUT1-positive area fraction. N = 9 M83 and n = 6 NTLs. CD31: Cluster of differentiation 31, DAPI: 4’,6-diamidino-2-phenylindole, GLUT1: Glucose transporter 1, NTL: non-transgenic littermate

**Fig. 6:**
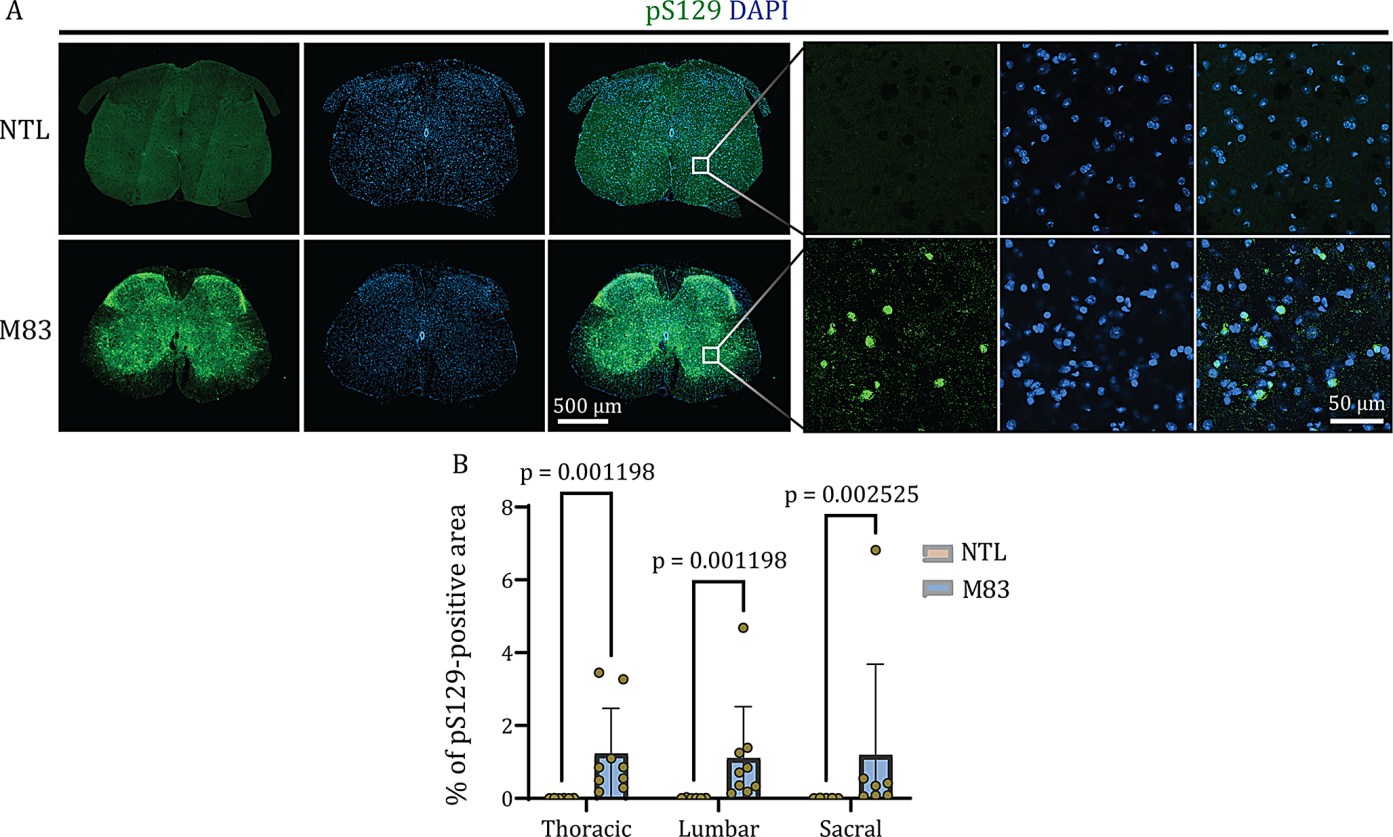
pS129 immunostaining in the spinal cords of M83 and NTL (control) mice: **(A)** Representative immunofluorescence images of colocalized pS129 (green) and DAPI (blue) showing pS129-positive (EP1536Y clone) signals at the lumbar level in the spinal cords of M83 mice compared to NTL mice; scale bars = 500 µm and 50 µm. **(B)** Quantitative evaluation of the pS129-positive areas in M83 and NTL mice at the level of the thoracic, lumbar, and sacral spinal cord segments. N = 9 M83 and n = 6 NTLs. DAPI: 4’,6-diamidino-2-phenylindole, NTL: non-transgenic littermate, pS129: α-syn phosphorylated at serine 129

Quantification using CSAs indicated that there was no volumetric alteration in the spinal cord GM or WM between M83 mice and their NTLs in any of the thoracic, lumbar, or sacral segments of the spinal cord or in the ratio of GM/WM (Fig. 4D). NeuN staining revealed an intact Clark’s column in the thoracic and lumbar spinal segments of M83 mice, although the number of motor neurons appeared to be relatively low (Supplemental Fig. 1A and B). We further examined whether there was atrophy in the brain of M83 mice using 9.4T MRI ex vivo. No structural abnormalities or volumetric alterations were observed in the M83 mice compared to NTL mice by using MRI (Supplemental Fig. 2A).

Earlier studies revealed a vicious circle between neuroinflammation and hypoxia, such as in multiple sclerosis [59], as well as in neurodegenerative diseases involving disrupted brain energy metabolism [60, 61]. Next, we performed staining for astrocytes (GFAP) and microglia (Iba1) in the different spinal segments of M83 mice. No apparent astrocytosis or microgliosis was observed throughout the spinal cord of M83 mice. Both astrocytes and microglia displayed homeostatic non-reactive morphology in the sections examined (Supplemental Fig. 1A and B and Supplemental Fig. 3A and B).

### Reduced sO_2_^SVOT^ in the spinal cord is not due to impaired vascular organization

To explore the cause of the reduction in sO_2_^SVOT^ in the spinal cord of M83 mice, we analyzed spinal vascular organization after in vivo imaging (Fig. 1C). We first performed immunostaining of the thoracic, lumbar, and sacral regions using the platelet endothelial cell adhesion molecule (CD31), which is expressed by differentiated endothelial cells (Fig. 5A) [51]. To assess the vasculature network, we used an ImageJ (Fiji) script to automatically calculate (1) the area fraction of blood vessels, (2) the length of blood vessels and, (3) the number of branches and junctions [57] based on the CD31 staining. No significant differences were detected in the vasculature area (*p* = 0.1586, *p* = 0.1827, and *p* = 0.1314), the number of branches (*p* = 0.3300, *p* = 0.3300, and *p* = 0.3127), or the vascular length (*p* = 0.2262, *p* = 0.1335, and *p* = 0.2001) in the thoracic, lumbar, and sacral segments between M83 and NTL control mice (Fig. 5B, C and D).

We then performed glucose transporter 1 (GLUT1) immunostaining to study the functionality of the vessel in the spinal cord. The GLUT1 protein is critical for transporting glucose across the blood‒brain barrier to the central nervous system and is highly expressed by endothelial cells [62]. The GLUT1-positive area fraction was not significantly different between M83 and NTL mice in any of the three spinal segments (*p* = 0.2559, *p* = 0.2559, and *p* = 0.3998, respectively, in the thoracic, lumbar and, sacral segments) (Fig. 5E). These results suggest that M83 mice do not exhibit vascular network disruption in the spinal cord.

### Αlpha-synuclein deposits throughout the spinal cord in M83 mice

Next, we assessed the distribution of α-syn pathology in the spinal cord sections of M83 and NTL mice using anti-pS129 α-syn antibodies. α-Syn undergoes posttranslational modifications, particularly phosphorylation at the Ser129 site [12, 13]. Given the complex findings reported in α-syn staining using different antibodies, we used three widely used antibodies against pS129 α-syn, i.e., the EP1536Y, 81A, and MJR-R13 clones. The pS129 α-syn signal (EP1536Y clone) was more abundant at the level of the thoracic (1.227% vs. 0.006%, *p* = 0.001198), lumbar (1.100% vs. 0.010%, *p* = 0.001198) and sacral (1.192% vs. 0.008%, *p* = 0.002525) spinal segments in M83 mice compared to NTLs, mainly in the GM and in the vicinity of the nuclei (Fig. 6A and B and Supplemental Fig. 4). Notably, a similar pS129 α-syn-positive signal was also detected in the brain, mainly in the cortex of M83 mice (Supplemental Fig. 2B). In contrast, the other two anti-pS129 α-syn antibodies, the 81A clone and, MJR-R13 clones presented reduced sensitivity, indicating differences in the signal intensity and localization of pS129-α-syn throughout the spinal cord of M83 mice in comparison to NTLs (Supplemental Fig. 5). Similar results in the spinal sO_2_^SVOT^, spinal volume and pS129 α-syn load were observed in M83 hetero- and homozygous mice.

## Discussion

In this work, we demonstrated the utility of SVOT in measuring reduced sO_2_^SVOT^ in the spinal cord of the M83 mouse model of α-synucleinopathy with sub-100-micron spatial resolution and unimpaired GM/WM structures by ex vivo T1w MRI, whereas ex vivo histological experiments revealed no vascular network dysfunction but greater pS129 α-syn accumulation in the spinal cord of M83 mice compared to NTL mice. To our knowledge, this work is the first to employ optoacoustic imaging and MRI for the spinal cord imaging in PD mouse model.

There are two data analysis tools developed and optimized in the current study, including (1) in-house deep learning-based model, based on a 3D nnUNet architecture, for automatic segmentation and quantification of mouse spinal T1w MR data for GM/WM volumetric analysis. Deep learning-based model trained on manually annotated slices and improved by active learning [52]. Tools for segmentation of human spinal cord was available in Spinal Cord Toolbox [54](v6.1), but not for mouse/rat spinal cord with different geometry, angles etc. We further validated the deep-learning analysis method with manual segmentation on another dataset. This open-source tool will be useful in future studies on mouse spinal cord structural MRI quantification. (2) an in-house pipeline to quantify the sO_2_^SVOT^ signal volumetrically in the spinal cord. We have previously quantified the oxygenation imaging in 2D using SVOT (sO_2_^SVOT^) in tumor of animal model with isoflurane anesthesia [33]. Thus far there is only one study using optoacoustics for in vivo measurement in the mouse spinal cord, of which the imaging was performed cross-sectionally [38]. Therefore the present study is the first for 3D imaging and quantification of oxygenation in mouse spinal cord, and in PD animal model in particular.

By using in vivo SVOT, we showed a 13–16% reduction in the sO_2_^SVOT^ level in the spinal cord of M83 mice compared with NTLs (under 1.5% inhalation isoflurane). Indeed, many studies have used optoacoustic/photoacoustic assessment of oxygenation with isoflurane anesthesia in the brain and trunk of mice. Isoflurane has varying effects on the hemoglobin oxygen dissociation curve in human blood samples at different doses [63]. Complicated factors may influence sO_2_ quantification, such as spectral coloring (wavelength-dependent attenuation) [64, 65], which can be partially compensated for by correcting for light attenuation with depth [66]. The measured sO_2_^SVOT^ might differ from the absolute value of oxygen saturation. Therefore, we used sO_2_^SVOT^ to represent a surrogate parameter for estimating relative changes in oxygen saturation. In addition, the time-to-anesthesia and the accumulated doses of isoflurane during the induction period differed among different mice. Our recording of time was not precise enough for calculating the accumulated isoflurane. Further studies are needed to investigate the effects of induction or accumulated doses of isoflurane on the in vivo measurement of sO_2_.

Reduced [^18^F]fluorodeoxyglucose uptake in the spinal cord has been observed in M83 mice at 9 months of age [27]. The low sO_2_^SVOT^ found was in line with previous studies on cerebral α-syn-associated mitochondrial degeneration in this model when treated with the pesticide paraquat, which is absent in the wild-type human α-syn M7 line [67]. In contrast, the administration of the iron chelator clioquinol, a compound that stabilizes functional hypoxia-inducible factor-1α, improved motor function in M83 mice [68]. The presence of solid bones and other acoustically mismatched tissues, such as the lungs, can lead to ultrasound aberrations, resulting in artifacts in optoacoustic imaging (e.g., the effects of large air-filled cavities [69]). The typical diameter of the spinal cord in mice is 2–3 mm [70], and the largest thickness of the vertebrae is approximately 200 μm. This is relatively thin compared with the acoustic wavelength for the central frequency of the array employed (215 μm). Therefore, the effects of acoustic aberrations are relatively small. In addition, in contrast to ultrasound imaging, the propagation of ultrasound waves in optoacoustic imaging is unidirectional, and consequently, aberrations are lower. Indeed, while we acknowledge that some distortion is produced by the vertebra in the spinal cord, several groups have reported full-body optoacoustic images of mice using transducers with frequencies of approximately 5–10 MHz, where the murine spinal cord could be accurately resolved by assuming a constant speed of sound [71–73]. The impact of mouse vertebrae on spectral unmixing does not result in significant changes in the propagation of light relative to that in other tissues. Potential acoustic distortions are the same for optoacoustic images at different wavelengths and hence have a limited impact on spectral unmixing. Indeed, we have shown that sO_2_ estimation is still possible through the human skull, which can induce strong acoustic aberrations [74]. The use of longer wavelengths (lower ultrasound frequencies) can reduce the aberrations associated with ultrasound propagation through a larger spinal cord. This, however, comes to the detriment of the achievable spatial resolution. We anticipate that an eventual clinical application of photoacoustics to image the spine would be based on relatively low frequencies of approximately 1 MHz (resolution around 1 mm) to avoid ultrasound aberrations, similar to transcranial clinical ultrasound applications. It is noted that the SVOT system was specifically designed to image mice/rats, and a different optoacoustic embodiment would be needed for clinical application in humans.

While functional and structural MRI has been performed on the spinal cord in wild-type [75, 76], tau [48], and amyotrophic lateral sclerosis [77, 78] mice. Very few in vivo or ex vivo MRI or optical studies have been performed in PD animal models [79, 80]. Our deep learning-based MRI assessment and NeuN staining did not reveal structural atrophy or apparent neuronal loss in the spinal cord of M83 mice compared with their NTLs. Low tissue oxygenation is known to contribute to neurodegeneration by reducing the energy supply to neurons in humans [81, 82]. Ealier studies showed that chronic cerebral hypoperfusion led to brain WM lesions [83] and neuronal functional deficits were associated with spinal cord hypoxia in an experimental autoimmune encephalomyelitis model [84]. Previous studies have shown that spinal axonal degeneration leads to myelin deterioration in 12-month-old M83 mice [41] and impaired neurons are found in the spinal cord of rodent models of 1-methyl-4-phenyl-1,2,3,6-tetrahydropyridine- or rotenone-induced experimental parkinsonism by immunostaining [85–87]. One possibility is that M83 mice exhibit oxygen saturation impairments in the spinal cord before presenting strong markers of neurodegeneration. In addition, ex vivo MRI might not be sensitive enough to detect axonal degeneration in the spinal cord of M83 mice at this age.

To understand the possible reasons for the observed reduction in sO_2_^SVOT^ in M83 compared with NTLs, we investigated alterations in the vasculature, glial activation, and α-syn levels. Our ex vivo analysis of spinal vascular organization and functionality suggested that the reduction in sO_2_^SVOT^ was not associated with vascular network impairment, as CD31 and GLUT1 expression did not significantly differ between the two strains. Hemodynamic changes are also possible under intact vascular structures via various microenvironmental mechanisms, including the control of the autonomic nervous system [88], which was not studied in the present study. In addition, the use of isoflurane has previously been shown to reduce the cerebral metabolic rate of oxygen and prevent hypoxia during cortical spreading depolarization in vitro [89]. An earlier study showed that isoflurane was able to modify the affinity between haemoglobin and oxygen by shifting the oxygen dissociation curve in human blood samples [63]. We did not measure the isoflurane concentration in the blood (e.g., through multiple gas chromatography‒mass spectrometry measurements), which could provide the ultimate quantification, owing to the limited amount of mouse blood and the complexity of the measurement. To minimize potential confounding effects due to anesthetics: We will focus on the following aspects: (1) awake mouse imaging: in vivo photoacoustic microscopy for hemodynamic and oxygen-metabolic responses has been demonstrated in the brain of awake mice in earlier studies [90]. However, this has not yet been implemented in our current imaging system for the mouse spinal cord. (2) standardized duration of anesthesia induction to reduce variability in the accumulated dose of anesthetics.3) Usage and comparison of alternative anesthetics with inhaled isoflurane anesthesia udsed in the current study. (3) minimizing dosage: Using the lowest effective dose of anesthesia will reduce physiological alterations while maintaining adequate immobilization. (4) continuous monitoring: By using a pulse oximeter to monitor oxygen saturation, blood pressure, and heart rate during anesthesia and imaging. This may help to detect any deviations caused by the anesthetic.

Neuroinflammation is closely linked to cerebral energy metabolism impairments in neurodegenerative diseases. Recently, the induction of neuroinflammation in a mouse model of Alzheimer’s disease was shown to elicit reductions in cerebral intravascular oxygen and increases in oxygen extraction in the brain [84, 91]. Neuroinflammatory pathology in an experimental autoimmune encephalomyelitis mouse model led to hypoxia accompanied by a reduction in spinal vascular perfusion [38]. In this study, we did not observe any signs of inflammation in the spinal cord of M83 mice, as astrocytes and microglia did not display reactive phenotypes. This finding is in line with an earlier study showing gliosis only in M83 and M83 mice injected with α-syn preformed fibrils [92]. 

pS129 α-syn is one of the most robust pathological markers of early α-syn aggregation, with almost all the aggregates containing this posttranslational modification [12, 13]. The accumulation of pS129 α-syn has also been observed in the context of oxygen intake and utilization disorders in cells, as well as in middle cerebral artery occlusion rodent models [20, 93], and in patients with obstructive sleep apnea syndrome [94]. Our study revealed an increase in the pS129 α-syn-positive (clone EP1536Y) signal within the spinal cord of M83 mice compared with their NTLs, where immunofluorescence staining revealed that the main location of pS129 α-syn was in the vicinity of the nucleus. Numerous antibodies targeting this posttranslational modification of α-syn have been developed with off-target effects, as well as non-specific binding and disparity in staining signals using these antibodies, as reported earlier [95–97]. It is noted that α-syn staining appeared to be an antibody (clone-dependent) in the spinal cord of M83 mice, with negative staining observed using two other clones targeting pS129 α-syn (81A and MJR-R13), which were positive for clone EP1536Y (which is robust and widely used) [95]. The difference in the pS129-positive signal observed and quantified between M83 mice and NTLs was genotype dependent in our study and was useful for studying early pathological changes in this model of PD. Further mechanistic studies on the potential association between low oxygen saturation and pS129 α-syn accumulation will be informative.

There are several limitations in the present study: (1) unmatched in vivo and ex vivo sample sizes and sex imbalance in the samples (mice with pigments on the skin inside the region of interest after shaving were excluded from the in vivo study and were only used for ex vivo analysis), also leading to small animal groups for in vivo SVOT; (2) Although the amount of inhaled isoflurane, concentration and flow rate during the imaging session were the same for all the mice, there was a variation in the time-to-anesthesia when the mice were under the anaesthesia induction. Therefore the accumulated dose of inhaled isoflurane in each mouse was not quantified and not compared between two groups, so a compounding effect of isoflurane on reduction of sO_2_ could not be excluded. Other factors such as partial pressure of oxygen, partial pressure of carbon dioxide, and pH also influence the sO_2_ [98], but were not measured in the current study. (3) The cervical part of the spine region displayed very little or no signal and thus could not be measured owing to the strong absorption of the BAT. (4) For logistic reasons, MRI was performed ex vivo. Since the goal of this imaging/observational study was to evaluate potential structural and oxygenation alterations in the spinal cord of a transgenic mouse model of PD, the mechanism for the observed reduction in the spinal oxygen concentration in M83 PD mice has not been clearly elucidated. Future studies are needed to address the underlying causes of the reduction in spinal oxygenation saturation and potential links between α-syn and oxygenation saturation in the spinal cord of M83 mice.

## Conclusion

In conclusion, we demonstrated the use of a non-invasive high-resolution volumetric imaging tool, SVOT, to quantify in vivo sO_2_^SVOT^ by using an in-house-developed analysis pipeline and deep learning-based automated quantification of volumetric MR images of the mouse spinal cord. We revealed reduced sO_2_^SVOT^ and pS129 α-syn accumulation, with no volumetric alterations, vascular impairments or inflammation in the spinal cord of PD M83 mice. The open-source automatic volumetric MRI analysis pipeline for the mouse spinal cord will be useful for applications in other models. These findings indicate the need for further research on α-syn-induced metabolic changes in the spinal cord.

## Electronic supplementary material

Below is the link to the electronic supplementary material.

Supplementary Material 1

Supplementary Material 2

Supplementary Material 3

Supplementary Material 4

Supplementary Material 5

Supplementary Material 6

## Data Availability

All raw data are available upon request to the corresponding authors. The code for deep learning-based WM and GM segmentation in T1W MR images is available at https://github.com/ivadomed/model_seg_mouse-sc_wm-gm_t1. The code for SVOT data analysis is available upon reasonable request.

## Abbreviations

α-syn: Alpha-synuclein
ANOVA: Analysis of variance
BAT: Brown adipose tissue
CD31: Cluster of differentiation 31
CSA: Cross-sectional area
DAPI: 4’,6-diamidino-2-phenylindole
GFAP: Glial fibrillary acidic protein
GLUT1: Glucose transporter 1
GM: Gray matter
HbO_2_: Oxygenated haemoglobin
HbR: Deoxygenated haemoglobin
MRI: Magnetic resonance imaging
NTL: Non-transgenic littermate
PBS: Phosphate-buffered saline
PD: Parkinson’s disease
pS129 α-syn: Phospho-α-syn (p-α-syn) at Ser129 site
ROI: Region of interest
sO_2_: Oxygen saturation
SVOT: Spiral volumetric optoacoustic tomography
T1w: T1-weighted
WM: White matter
SNCA: Human alpha-synuclein gene

## References

[1] Global regional. Lancet Neurol. 2018;17:939–53. 10.1016/s1474-4422(18)30295-3. and national burden of Parkinson’s disease, 1990–2016: a systematic analysis for the Global Burden of Disease Study 2016. 10.1016/S1474-4422(18)30295-3 PMC6191528 30287051

[2] Guo M, Ji X, Liu J. Hypoxia and Alpha-Synuclein: Inextricable Link underlying the pathologic progression of Parkinson’s Disease. Front Aging Neurosci. 2022;14:919343. 10.3389/fnagi.2022.919343. 35959288 10.3389/fnagi.2022.919343 PMC9360429

[3] Lestón Pinilla L, Ugun-Klusek A, Rutella S, De Girolamo LA. Hypoxia Signaling in Parkinson’s Disease: There Is Use in Asking What HIF? Biology (Basel). 2021;10. 10.3390/biology10080723. 10.3390/biology10080723 PMC8389254 34439955

[4] Melzer TR, Watts R, MacAskill MR, Pearson JF, Rüeger S, Pitcher TL, et al. Arterial spin labelling reveals an abnormal cerebral perfusion pattern in Parkinson’s disease. Brain. 2011;134:845–55. 10.1093/brain/awq377. 21310726 10.1093/brain/awq377 PMC3105489

[5] Pang SY-Y, Ho PW-L, Liu H-F, Leung C-T, Li L, Chang EES, et al. The interplay of aging, genetics and environmental factors in the pathogenesis of Parkinson’s disease. Translational Neurodegeneration. 2019;8:23. 10.1186/s40035-019-0165-9. 31428316 10.1186/s40035-019-0165-9 PMC6696688

[6] Blauwendraat C, Nalls MA, Singleton AB. The genetic architecture of Parkinson’s disease. Lancet Neurol. 2020;19:170–8. 10.1016/s1474-4422(19)30287-x. 31521533 10.1016/S1474-4422(19)30287-X PMC8972299

[7] Murata H, Barnhill LM, Bronstein JM. Air Pollution and the risk of Parkinson’s disease: a review. Mov Disord. 2022;37:894–904. 10.1002/mds.28922. 35043999 10.1002/mds.28922 PMC9119911

[8] Hatcher JM, Pennell KD, Miller GW. Parkinson’s disease and pesticides: a toxicological perspective. Trends Pharmacol Sci. 2008;29:322–9. 10.1016/j.tips.2008.03.007. 18453001 10.1016/j.tips.2008.03.007 PMC5683846

[9] Burtscher J, Duderstadt Y, Gatterer H, Burtscher M, Vozdek R, Millet GP, et al. Hypoxia Sensing and Responses in Parkinson’s Disease. Int J Mol Sci. 2024;25. 10.3390/ijms25031759. 10.3390/ijms25031759 PMC10855464 38339038

[10] Visser AE, de Vries NM, Richard E, Bloem BR. Tackling vascular risk factors as a possible disease modifying intervention in Parkinson’s disease. NPJ Parkinsons Dis. 2024;10:50. 10.1038/s41531-024-00666-6. 38431725 10.1038/s41531-024-00666-6 PMC10908840

[11] Kummer BR, Diaz I, Wu X, Aaroe AE, Chen ML, Iadecola C, et al. Associations between cerebrovascular risk factors and parkinson disease. Ann Neurol. 2019;86:572–81. 10.1002/ana.25564. 31464350 10.1002/ana.25564 PMC6951811

[12] Fujiwara H, Hasegawa M, Dohmae N, Kawashima A, Masliah E, Goldberg MS, et al. Alpha-synuclein is phosphorylated in synucleinopathy lesions. Nat Cell Biol. 2002;4:160–4. 10.1038/ncb748. 11813001 10.1038/ncb748

[13] Anderson JP, Walker DE, Goldstein JM, de Laat R, Banducci K, Caccavello RJ, et al. Phosphorylation of Ser-129 is the dominant pathological modification of alpha-synuclein in familial and sporadic Lewy body disease. J Biol Chem. 2006;281:29739–52. 10.1074/jbc.M600933200. 16847063 10.1074/jbc.M600933200

[14] Braak H, Del Tredici K, Rüb U, de Vos RA, Jansen Steur EN, Braak E. Staging of brain pathology related to sporadic Parkinson’s disease. Neurobiol Aging. 2003;24:197–211. 10.1016/s0197-4580(02)00065-9. 12498954 10.1016/s0197-4580(02)00065-9

[15] Landelle C, Dahlberg LS, Lungu O, Misic B, De Leener B, Doyon J. Altered Spinal Cord Functional Connectivity Associated with Parkinson’s Disease Progression. Mov Disord. 2023;38:636–45. 10.1002/mds.29354. 36802374 10.1002/mds.29354

[16] Braak H, Del Tredici K. Neuropathological staging of Brain Pathology in sporadic Parkinson’s disease: separating the wheat from the Chaff. J Parkinsons Dis. 2017;7:S71–85. 10.3233/jpd-179001. 28282810 10.3233/JPD-179001 PMC5345633

[17] Del Tredici K, Braak H. Spinal cord lesions in sporadic Parkinson’s disease. Acta Neuropathol. 2012;124:643–64. 10.1007/s00401-012-1028-y. 22926675 10.1007/s00401-012-1028-y

[18] Guo M, Liu W, Luo H, Shao Q, Li Y, Gu Y, et al. Hypoxic stress accelerates the propagation of pathological alpha-synuclein and degeneration of dopaminergic neurons. CNS Neurosci Ther. 2023;29:544–58. 10.1111/cns.14055. 36514210 10.1111/cns.14055 PMC9873519

[19] Li G, Liu J, Guo M, Gu Y, Guan Y, Shao Q, et al. Chronic hypoxia leads to cognitive impairment by promoting HIF-2α-mediated ceramide catabolism and alpha-synuclein hyperphosphorylation. Cell Death Discovery. 2022;8:473. 10.1038/s41420-022-01260-6. 36450714 10.1038/s41420-022-01260-6 PMC9712431

[20] Kim T, Mehta SL, Kaimal B, Lyons K, Dempsey RJ, Vemuganti R. Poststroke induction of α-Synuclein mediates ischemic brain damage. J Neurosci. 2016;36:7055–65. 10.1523/jneurosci.1241-16.2016. 27358461 10.1523/JNEUROSCI.1241-16.2016 PMC4994709

[21] Sun HL, Sun BL, Chen DW, Chen Y, Li WW, Xu MY, et al. Plasma α-synuclein levels are increased in patients with obstructive sleep apnea syndrome. Ann Clin Transl Neurol. 2019;6:788–94. 10.1002/acn3.756. 31020003 10.1002/acn3.756 PMC6469340

[22] Meyer BP, Hirschler L, Lee S, Kurpad SN, Warnking JM, Barbier EL, et al. Optimized cervical spinal cord perfusion MRI after traumatic injury in the rat. J Cereb Blood Flow Metab. 2021;41:2010–25. 10.1177/0271678x20982396. 33509036 10.1177/0271678X20982396 PMC8327111

[23] Matsubayashi K, Nagoshi N, Komaki Y, Kojima K, Shinozaki M, Tsuji O, et al. Assessing cortical plasticity after spinal cord injury by using resting-state functional magnetic resonance imaging in awake adult mice. Sci Rep. 2018;8:14406. 10.1038/s41598-018-32766-8. 30258091 10.1038/s41598-018-32766-8 PMC6158265

[24] Laakso H, Lehto LJ, Paasonen J, Salo R, Canna A, Lavrov I, et al. Spinal cord fMRI with MB-SWIFT for assessing epidural spinal cord stimulation in rats. Magn Reson Med. 2021;86:2137–45. 10.1002/mrm.28844. 34002880 10.1002/mrm.28844 PMC8360072

[25] Wu W, He S, Wu J, Chen C, Li X, Liu K, et al. Long-term in vivo imaging of mouse spinal cord through an optically cleared intervertebral window. Nat Commun. 2022;13:1959. 10.1038/s41467-022-29496-x. 35414131 10.1038/s41467-022-29496-x PMC9005710

[26] Esipova TV, Barrett MJP, Erlebach E, Masunov AE, Weber B, Vinogradov SA. Oxyphor 2P: a high-performance probe for deep-tissue longitudinal oxygen imaging. Cell Metab. 2019;29:736–e447. 10.1016/j.cmet.2018.12.022. 30686745 10.1016/j.cmet.2018.12.022 PMC6402963

[27] Mondal R, Campoy A-DT, Liang C, Mukherjee J. [18F]FDG PET/CT studies in transgenic hualpha-syn (A53T) Parkinson’s Disease Mouse Model of α-Synucleinopathy. Front NeuroSci. 2021;15:718. 10.3389/fnins.2021.676257 PMC8239288 34211366

[28] Harmon JN, Chandran P, Chandrasekaran A, Hyde JE, Hernandez GJ, Reed MJ, et al. Contrast-enhanced ultrasound imaging detects anatomical and functional changes in rat cervical spine microvasculature with normal aging. bioRxiv. 2024. 10.1101/2024.03.12.584672. 39188137 10.1093/gerona/glae215 PMC11701746

[29] Claron J, Hingot V, Rivals I, Rahal L, Couture O, Deffieux T, et al. Large-scale functional ultrasound imaging of the spinal cord reveals in-depth spatiotemporal responses of spinal nociceptive circuits in both normal and inflammatory states. Pain. 2021;162:1047–59. 10.1097/j.pain.0000000000002078. 32947542 10.1097/j.pain.0000000000002078 PMC7977620

[30] Ni R, Rudin M, Klohs J. Cortical hypoperfusion and reduced cerebral metabolic rate of oxygen in the arcAβ mouse model of Alzheimer’s disease. Photoacoustics. 2018;10:38–47. 10.1016/j.pacs.2018.04.001. 29682448 10.1016/j.pacs.2018.04.001 PMC5909030

[31] Deán-Ben XL, Robin J, Nozdriukhin D, Ni R, Zhao J, Glück C, et al. Deep optoacoustic localization microangiography of ischemic stroke in mice. Nat Commun. 2023;14:3584. 10.1038/s41467-023-39069-1. 37328490 10.1038/s41467-023-39069-1 PMC10275987

[32] Vaas M, Ni R, Rudin M, Kipar A, Klohs J. Extracerebral tissue damage in the Intraluminal Filament Mouse Model of Middle Cerebral Artery Occlusion. Front Neurol. 2017;8:85. 10.3389/fneur.2017.00085. 28348545 10.3389/fneur.2017.00085 PMC5347084

[33] Ron A, Deán-Ben XL, Gottschalk S, Razansky D. Volumetric optoacoustic imaging unveils high-resolution patterns of Acute and cyclic hypoxia in a murine model of breast Cancer. Cancer Res. 2019;79:4767–75. 10.1158/0008-5472.can-18-3769. 31097477 10.1158/0008-5472.CAN-18-3769

[34] Ni R, Chen Z, Deán-Ben XL, Voigt FF, Kirschenbaum D, Shi G, et al. Multiscale optical and optoacoustic imaging of amyloid-β deposits in mice. Nat Biomedical Eng. 2022. 10.1038/s41551-022-00906-1. 10.1038/s41551-022-00906-1 35835994

[35] Vagenknecht P, Luzgin A, Ono M, Ji B, Higuchi M, Noain D, et al. Non-invasive imaging of tau-targeted probe uptake by whole brain multi-spectral optoacoustic tomography. Eur J Nucl Med Mol Imaging. 2022. 10.1007/s00259-022-05708-w. 35128565 10.1007/s00259-022-05708-w PMC9165274

[36] Ni R, Straumann N, Fazio S, Dean-Ben XL, Louloudis G, Keller C, et al. Imaging increased metabolism in the spinal cord in mice after middle cerebral artery occlusion. Photoacoustics. 2023;32:100532. 10.1016/j.pacs.2023.100532. 37645255 10.1016/j.pacs.2023.100532 PMC10461215

[37] Straumann N, Combes BF, Dean Ben XL, Sternke-Hoffmann R, Gerez JA, Dias I, et al. Visualizing alpha-synuclein and iron deposition in M83 mouse model of Parkinson’s disease in vivo. Brain Pathol. 2024;e13288. 10.1111/bpa.13288. 10.1111/bpa.13288 PMC11483525 38982662

[38] Ramos-Vega M, Kjellman P, Todorov MI, Kylkilahti TM, Bäckström BT, Ertürk A, et al. Mapping of neuroinflammation-induced hypoxia in the spinal cord using optoacoustic imaging. Acta Neuropathol Commun. 2022;10:51. 10.1186/s40478-022-01337-4. 35410629 10.1186/s40478-022-01337-4 PMC8996517

[39] Kalva SK, Deán-Ben XL, Reiss M, Razansky D. Spiral volumetric optoacoustic tomography for imaging whole-body biodynamics in small animals. Nat Protoc. 2023;18:2124–42. 10.1038/s41596-023-00834-7. 37208409 10.1038/s41596-023-00834-7

[40] Kalva SK, Deán-Ben XL, Reiss M, Razansky D. Head-to-tail imaging of mice with spiral volumetric optoacoustic tomography. Photoacoustics. 2023;30:100480. 10.1016/j.pacs.2023.100480. 37025111 10.1016/j.pacs.2023.100480 PMC10070820

[41] Giasson BI, Duda JE, Quinn SM, Zhang B, Trojanowski JQ, Lee VM. Neuronal alpha-synucleinopathy with severe movement disorder in mice expressing A53T human alpha-synuclein. Neuron. 2002;34:521–33. 10.1016/s0896-6273(02)00682-7. 12062037 10.1016/s0896-6273(02)00682-7

[42] Kalva SK, Sánchez-Iglesias A, Deán-Ben XL, Liz-Marzán LM, Razansky D. Rapid Volumetric Optoacoustic Tracking of Nanoparticle Kinetics across Murine organs. ACS Appl Mater Interfaces. 2022;14:172–8. 10.1021/acsami.1c17661. 34949083 10.1021/acsami.1c17661

[43] Ron A, Kalva SK, Periyasamy V, Deán-Ben XL, Razansky D. Biomedical Imaging: Flash Scanning Volumetric Optoacoustic Tomography for High Resolution Whole-Body Tracking of Nanoagent Kinetics and Biodistribution (Laser Photonics Rev. 15(3)/2021). Laser & Photonics Reviews. 2021;15:2170021. 10.1002/lpor.202170021

[44] Benskey MJ, Perez RG, Manfredsson FP. The contribution of alpha synuclein to neuronal survival and function - implications for Parkinson’s disease. J Neurochem. 2016;137:331–59. 10.1111/jnc.13570. 26852372 10.1111/jnc.13570 PMC5021132

[45] Jacques SL. Optical properties of biological tissues: a review. Phys Med Biol. 2013;58:R37–61. 10.1088/0031-9155/58/11/r37. 23666068 10.1088/0031-9155/58/11/R37

[46] American National Standards I. Laser Institute of A. ANSI Z136.1 Safe Use of lasers – 2022. Laser Institute of America; 2022.

[47] Watson C, Paxinos G, Kayalioglu G, Heise C. Chapter 16 - atlas of the mouse spinal cord. In: Watson C, Paxinos G, Kayalioglu G, editors. The spinal cord. San Diego: Academic; 2009. pp. 308–79.

[48] Sartoretti T, Ganley RP, Ni R, Freund P, Zeilhofer HU, Klohs J. Structural MRI reveals cervical spinal cord atrophy in the P301L mouse model of Tauopathy: gender and transgene-dosing effects. Front Aging Neurosci. 2022;14. 10.3389/fnagi.2022.825996 PMC9108240 35585865

[49] Massalimova A, Ni R, Nitsch RM, Reisert M, von Elverfeldt D, Klohs J. Diffusion Tensor Imaging reveals whole-brain microstructural changes in the P301L mouse model of Tauopathy. Neurodegener Dis. 2021;1–12. 10.1159/000515754. 10.1159/000515754 33975312

[50] Ni R, Zarb Y, Kuhn GA, Müller R, Yundung Y, Nitsch RM, et al. SWI and phase imaging reveal intracranial calcifications in the P301L mouse model of human tauopathy. Magma. 2020;33:769–81. 10.1007/s10334-020-00855-3. 32468149 10.1007/s10334-020-00855-3 PMC7669813

[51] Kecheliev V, Boss L, Maheshwari U, Konietzko U, Keller A, Razansky D, et al. Aquaporin 4 is differentially increased and dislocated in association with tau and amyloid-beta. Life Sci. 2023;121593. 10.1016/j.lfs.2023.121593. 10.1016/j.lfs.2023.121593 36934970

[52] Cohen-Adad J. Segmentation model of ex vivo mouse spinal cord white and gray matter. Zenodo; 2024.

[53] Isensee F, Jaeger PF, Kohl SAA, Petersen J, Maier-Hein KH. nnU-Net: a self-configuring method for deep learning-based biomedical image segmentation. Nat Methods. 2021;18:203–11. 10.1038/s41592-020-01008-z. 33288961 10.1038/s41592-020-01008-z

[54] De Leener B, Lévy S, Dupont SM, Fonov VS, Stikov N, Louis Collins D, et al. SCT: spinal cord toolbox, an open-source software for processing spinal cord MRI data. NeuroImage. 2017;145:24–43. 10.1016/j.neuroimage.2016.10.009. 27720818 10.1016/j.neuroimage.2016.10.009

[55] Fiederling F, Hammond LA, Ng D, Mason C, Dodd J. SpineRacks and SpinalJ for efficient analysis of neurons in a 3D reference atlas of the mouse spinal cord. STAR Protoc. 2021;2:100897. 10.1016/j.xpro.2021.100897. 34841273 10.1016/j.xpro.2021.100897 PMC8605391

[56] Sobek J, Li J, Combes BF, Gerez JA, Henrich MT, Geibl FF, et al. Efficient characterization of multiple binding sites of small molecule imaging ligands on amyloid-beta, tau and alpha-synuclein. Eur J Nucl Med Mol Imaging. 2024. 10.1007/s00259-024-06806-7. 38953933 10.1007/s00259-024-06806-7 PMC11527973

[57] Rust R, Grönnert L, Gantner C, Enzler A, Mulders G, Weber RZ, et al. Nogo-A targeted therapy promotes vascular repair and functional recovery following stroke. Proc Natl Acad Sci U S A. 2019;116:14270–9. 10.1073/pnas.1905309116. 31235580 10.1073/pnas.1905309116 PMC6628809

[58] Reber J, Willershäuser M, Karlas A, Paul-Yuan K, Diot G, Franz D, et al. Non-invasive measurement of Brown Fat Metabolism based on Optoacoustic Imaging of Hemoglobin Gradients. Cell Metab. 2018;27:689–e7014. 10.1016/j.cmet.2018.02.002. 29514074 10.1016/j.cmet.2018.02.002

[59] Yang R, Dunn JF. Multiple sclerosis disease progression: contributions from a hypoxia-inflammation cycle. Mult Scler. 2019;25:1715–8. 10.1177/1352458518791683. 30052113 10.1177/1352458518791683 PMC6826859

[60] Butovsky O, Weiner HL. Microglial signatures and their role in health and disease. Nat Rev Neurosci. 2018;19:622–35. 10.1038/s41583-018-0057-5. 30206328 10.1038/s41583-018-0057-5 PMC7255106

[61] van Horssen J, van Schaik P, Witte M. Inflammation and mitochondrial dysfunction: a vicious circle in neurodegenerative disorders? Neurosci Lett. 2019;710:132931. 10.1016/j.neulet.2017.06.050. 28668382 10.1016/j.neulet.2017.06.050

[62] Kong Y, Maschio CA, Shi X, Xie F, Zuo C, Konietzko U, et al. Relationship between reactive astrocytes, by [(18)F]SMBT-1 imaging, with Amyloid-Beta, tau, glucose metabolism, and TSPO in mouse models of Alzheimer’s Disease. Mol Neurobiol. 2024. 10.1007/s12035-024-04106-7. 38502413 10.1007/s12035-024-04106-7 PMC11415417

[63] Ronzani M, Woyke S, Mair N, Gatterer H, Oberacher H, Plunser D, et al. The effect of desflurane, isoflurane and sevoflurane on the hemoglobin oxygen dissociation curve in human blood samples. Sci Rep. 2022;12:13633. 10.1038/s41598-022-17789-6. 35948604 10.1038/s41598-022-17789-6 PMC9365211

[64] Hochuli R, An L, Beard PC, Cox BT. Estimating blood oxygenation from photoacoustic images: can a simple linear spectroscopic inversion ever work? J Biomed Opt. 2019;24:1–13. 10.1117/1.jbo.24.12.121914. 31849203 10.1117/1.JBO.24.12.121914 PMC7005536

[65] Cox B, Laufer JG, Arridge SR, Beard PC. Quantitative spectroscopic photoacoustic imaging: a review. J Biomed Opt. 2012;17:061202. 10.1117/1.jbo.17.6.061202. 22734732 10.1117/1.JBO.17.6.061202

[66] Chen Z, Zhou Q, Deán-Ben XL, Gezginer I, Ni R, Reiss M, et al. Multimodal Noninvasive Functional Neurophotonic imaging of murine brain-wide sensory responses. Adv Sci (Weinh). 2022;9:e2105588. 10.1002/advs.202105588. 35798308 10.1002/advs.202105588 PMC9404388

[67] Norris EH, Uryu K, Leight S, Giasson BI, Trojanowski JQ, Lee VM. Pesticide exposure exacerbates alpha-synucleinopathy in an A53T transgenic mouse model. Am J Pathol. 2007;170:658–66. 10.2353/ajpath.2007.060359. 17255333 10.2353/ajpath.2007.060359 PMC1851868

[68] Finkelstein DI, Hare DJ, Billings JL, Sedjahtera A, Nurjono M, Arthofer E, et al. Clioquinol improves cognitive, motor function, and Microanatomy of the alpha-synuclein hA53T transgenic mice. ACS Chem Neurosci. 2016;7:119–29. 10.1021/acschemneuro.5b00253. 26481462 10.1021/acschemneuro.5b00253

[69] Dean-Ben XL, Ma R, Razansky D, Ntziachristos V. Statistical approach for optoacoustic image reconstruction in the presence of strong acoustic heterogeneities. IEEE Trans Med Imaging. 2011;30:401–8. 10.1109/tmi.2010.2081683. 20876007 10.1109/TMI.2010.2081683

[70] Ong HH, Wehrli FW. Quantifying axon diameter and intra-cellular volume fraction in excised mouse spinal cord with q-space imaging. NeuroImage. 2010;51:1360–6. 10.1016/j.neuroimage.2010.03.063. 20350604 10.1016/j.neuroimage.2010.03.063 PMC2895496

[71] Merčep E, Herraiz JL, Deán-Ben XL, Razansky D. Transmission–reflection optoacoustic ultrasound (TROPUS) computed tomography of small animals. Light: Sci Appl. 2019;8:18. 10.1038/s41377-019-0130-5. 30728957 10.1038/s41377-019-0130-5 PMC6351605

[72] Choi S, Yang J, Lee SY, Kim J, Lee J, Kim WJ, et al. Deep learning enhances multiparametric dynamic volumetric photoacoustic computed tomography in vivo (DL-PACT). Adv Sci (Weinh). 2022;10:e2202089. 10.1002/advs.202202089. 36354200 10.1002/advs.202202089 PMC9811490

[73] Asao Y, Nagae K, Miyasaka K, Sekiguchi H, Aiso S, Watanabe S, et al. In vivo label-free Observation of Tumor-related blood vessels in small animals using a newly designed photoacoustic 3D imaging system. Ultrason Imaging. 2022;44:96–104. 10.1177/01617346221099201. 35549598 10.1177/01617346221099201 PMC9207988

[74] Ni R, Deán-Ben XL, Treyer V, Gietl A, Hock C, Klohs J, et al. Coregistered transcranial optoacoustic and magnetic resonance angiography of the human brain. Opt Lett. 2023;48:648–51. 10.1364/OL.475578. 36723554 10.1364/OL.475578

[75] Saito S, Mori Y, Yoshioka Y, Murase K. High-resolution ex vivo imaging in mouse spinal cord using micro-CT with 11.7T-MRI and myelin staining validation. Neurosci Res. 2012;73:337–40. 10.1016/j.neures.2012.05.004. 22609867 10.1016/j.neures.2012.05.004

[76] Bilgen M, Al-Hafez B, Berman NE, Festoff BW. Magnetic resonance imaging of mouse spinal cord. Magn Reson Med. 2005;54:1226–31. 10.1002/mrm.20672. 16206177 10.1002/mrm.20672

[77] Gao J, Jiang M, Magin RL, Gatto RG, Morfini G, Larson AC, et al. Multicomponent diffusion analysis reveals microstructural alterations in spinal cord of a mouse model of amyotrophic lateral sclerosis ex vivo. PLoS ONE. 2020;15:e0231598. 10.1371/journal.pone.0231598. 32310954 10.1371/journal.pone.0231598 PMC7170503

[78] Gatto RG, Li W, Gao J, Magin RL. In vivo diffusion MRI detects early spinal cord axonal pathology in a mouse model of amyotrophic lateral sclerosis. NMR Biomed. 2018;31:e3954. 10.1002/nbm.3954. 30117615 10.1002/nbm.3954

[79] Ni R. PET imaging in animal models of Parkinson’s disease. Behav Brain Res. 2022;114174. 10.1016/j.bbr.2022.114174. 10.1016/j.bbr.2022.114174 36283568

[80] Chu WT, DeSimone JC, Riffe CJ, Liu H, Chakrabarty P, Giasson BI, et al. α-Synuclein induces progressive changes in Brain microstructure and sensory-evoked brain function that precedes locomotor decline. J Neurosci. 2020;40:6649–59. 10.1523/jneurosci.0189-20.2020. 32669353 10.1523/JNEUROSCI.0189-20.2020 PMC7486650

[81] Hernandez-Gerez E, Fleming IN, Parson SH. A role for spinal cord hypoxia in neurodegeneration. Cell Death Dis. 2019;10:861. 10.1038/s41419-019-2104-1. 31723121 10.1038/s41419-019-2104-1 PMC6853899

[82] Wolters FJ, Zonneveld HI, Hofman A, van der Lugt A, Koudstaal PJ, Vernooij MW, et al. Cerebral perfusion and the risk of dementia: a Population-based study. Circulation. 2017;136:719–28. 10.1161/circulationaha.117.027448. 28588075 10.1161/CIRCULATIONAHA.117.027448

[83] Shibata M, Ohtani R, Ihara M, Tomimoto H. White matter lesions and glial activation in a novel mouse model of chronic cerebral hypoperfusion. Stroke. 2004;35:2598–603. 10.1161/01.str.0000143725.19053.60. 15472111 10.1161/01.STR.0000143725.19053.60

[84] Davies AL, Desai RA, Bloomfield PS, McIntosh PR, Chapple KJ, Linington C, et al. Neurological deficits caused by tissue hypoxia in neuroinflammatory disease. Ann Neurol. 2013;74:815–25. 10.1002/ana.24006. 24038279 10.1002/ana.24006

[85] Chera B, Schaecher KE, Rocchini A, Imam SZ, Ray SK, Ali SF, et al. Calpain upregulation and neuron death in spinal cord of MPTP-induced parkinsonism in mice. Ann N Y Acad Sci. 2002;965:274–80. 10.1111/j.1749-6632.2002.tb04169.x. 12105103 10.1111/j.1749-6632.2002.tb04169.x

[86] Chera B, Schaecher KE, Rocchini A, Imam SZ, Sribnick EA, Ray SK, et al. Immunofluorescent labeling of increased calpain expression and neuronal death in the spinal cord of 1-methyl-4-phenyl-1,2,3,6-tetrahydropyridine-treated mice. Brain Res. 2004;1006:150–6. 10.1016/j.brainres.2004.01.065. 15051518 10.1016/j.brainres.2004.01.065

[87] Samantaray S, Knaryan VH, Guyton MK, Matzelle DD, Ray SK, Banik NL. The parkinsonian neurotoxin rotenone activates calpain and caspase-3 leading to motoneuron degeneration in spinal cord of Lewis rats. Neuroscience. 2007;146:741–55. 10.1016/j.neuroscience.2007.01.056. 17367952 10.1016/j.neuroscience.2007.01.056 PMC1940329

[88] Koep JL, Taylor CE, Coombes JS, Bond B, Ainslie PN, Bailey TG. Autonomic control of cerebral blood flow: fundamental comparisons between peripheral and cerebrovascular circulations in humans. J Physiol. 2022;600:15–39. 10.1113/jp281058. 34842285 10.1113/JP281058

[89] Schoknecht K, Maechler M, Wallach I, Dreier JP, Liotta A, Berndt N. Isoflurane lowers the cerebral metabolic rate of oxygen and prevents hypoxia during cortical spreading depolarization in vitro: an integrative experimental and modeling study. J Cereb Blood Flow Metab. 2024;44:1000–12. 10.1177/0271678x231222306. 38140913 10.1177/0271678X231222306 PMC11318408

[90] Cao R, Tran A, Li J, Xu Z, Sun N, Zuo Z, et al. Hemodynamic and oxygen-metabolic responses of the awake mouse brain to hypercapnia revealed by multi-parametric photoacoustic microscopy. J Cereb Blood Flow Metab. 2021;41:2628–39. 10.1177/0271678x211010352. 33899557 10.1177/0271678X211010352 PMC8504963

[91] Liu C, Cárdenas-Rivera A, Teitelbaum S, Birmingham A, Alfadhel M, Yaseen MA. Neuroinflammation increases oxygen extraction in a mouse model of Alzheimer’s disease. Alzheimers Res Ther. 2024;16:78. 10.1186/s13195-024-01444-5. 38600598 10.1186/s13195-024-01444-5 PMC11005245

[92] Sorrentino ZA, Xia Y, Funk C, Riffe CJ, Rutherford NJ, Ceballos Diaz C, et al. Motor neuron loss and neuroinflammation in a model of α-synuclein-induced neurodegeneration. Neurobiol Dis. 2018;120:98–106. 10.1016/j.nbd.2018.09.005. 30195075 10.1016/j.nbd.2018.09.005 PMC6190709

[93] Unal-Cevik I, Gursoy-Ozdemir Y, Yemisci M, Lule S, Gurer G, Can A, et al. Alpha-synuclein aggregation induced by brief ischemia negatively impacts neuronal survival in vivo: a study in [A30P]alpha-synuclein transgenic mouse. J Cereb Blood Flow Metab. 2011;31:913–23. 10.1038/jcbfm.2010.170. 20877387 10.1038/jcbfm.2010.170 PMC3063624

[94] Chen T, Li J, Chao D, Sandhu HK, Liao X, Zhao J, et al. δ-Opioid receptor activation reduces α-synuclein overexpression and oligomer formation induced by MPP(+) and/or hypoxia. Exp Neurol. 2014;255:127–36. 10.1016/j.expneurol.2014.02.022. 24613828 10.1016/j.expneurol.2014.02.022

[95] Delic V, Chandra S, Abdelmotilib H, Maltbie T, Wang S, Kem D, et al. Sensitivity and specificity of phospho-Ser129 α-synuclein monoclonal antibodies. J Comp Neurol. 2018;526:1978–90. 10.1002/cne.24468. 29888794 10.1002/cne.24468 PMC6031478

[96] Lashuel HA, Mahul-Mellier AL, Novello S, Hegde RN, Jasiqi Y, Altay MF, et al. Revisiting the specificity and ability of phospho-S129 antibodies to capture alpha-synuclein biochemical and pathological diversity. NPJ Parkinsons Dis. 2022;8:136. 10.1038/s41531-022-00388-7. 36266318 10.1038/s41531-022-00388-7 PMC9584898

[97] Kumar ST, Jagannath S, Francois C, Vanderstichele H, Stoops E, Lashuel HA. How specific are the conformation-specific α-synuclein antibodies? Characterization and validation of 16 α-synuclein conformation-specific antibodies using well-characterized preparations of α-synuclein monomers, fibrils and oligomers with distinct structures and morphology. Neurobiol Dis. 2020;146:105086. 10.1016/j.nbd.2020.105086. 32971232 10.1016/j.nbd.2020.105086

[98] Colby LA, Morenko BJ. Clinical considerations in rodent bioimaging. Comp Med. 2004;54:623–30. 15679259

