# Supplementary figures and images for: Spiral volumetric optoacoustic tomography of reduced oxygen saturation in the spinal cord of M83 mouse model of Parkinson’s disease
Source: Eur J Nucl Med Mol Imaging. 2024 Oct 9;52(2):427–43. doi: 10.1007/s00259-024-06938-w (PMC11732882; doi:10.1007/s00259-024-06938-w)

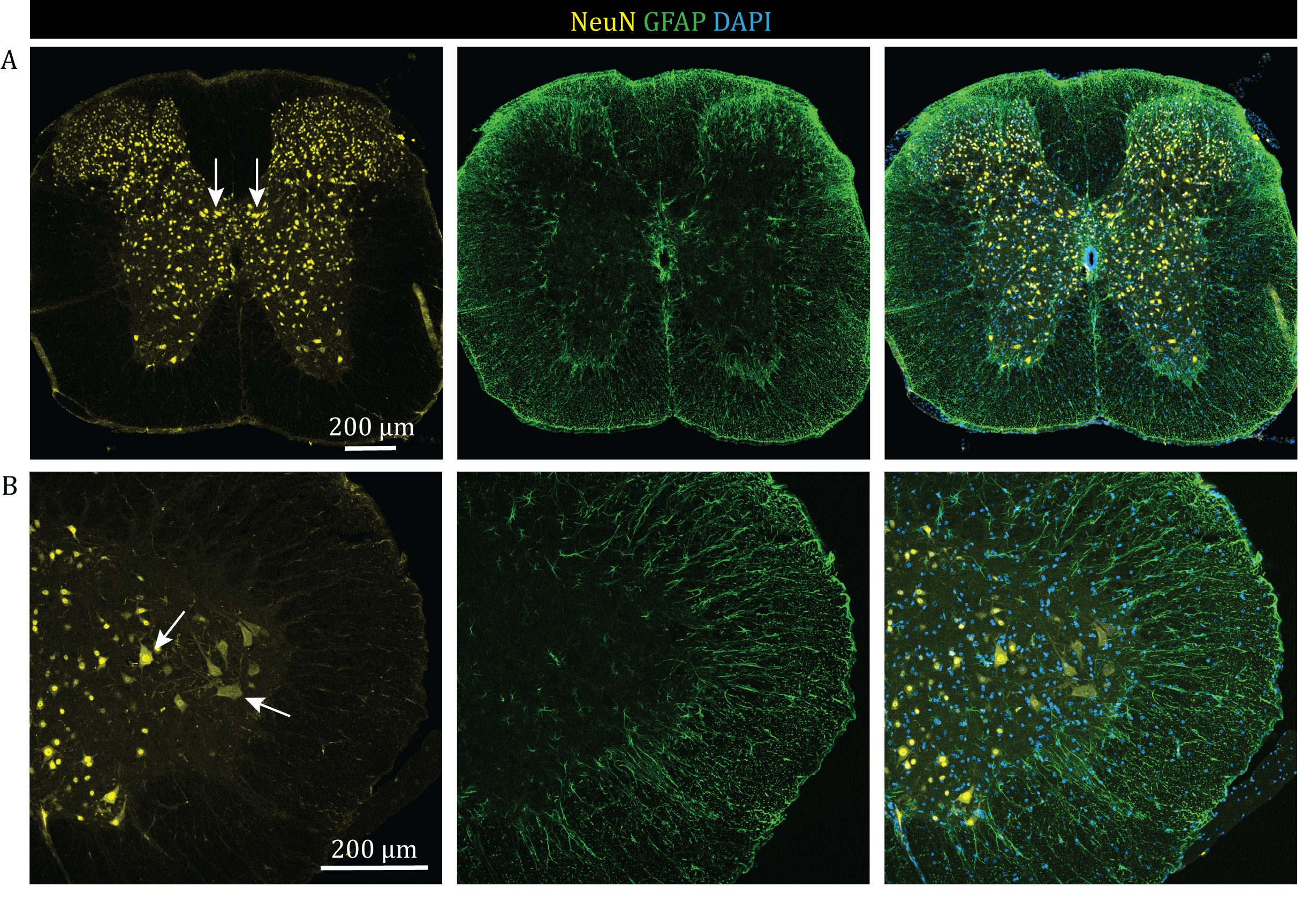

Supplement: Supplementary file 1 — Supplementary Material 1 [file 259_2024_6938_MOESM1_ESM.tif]

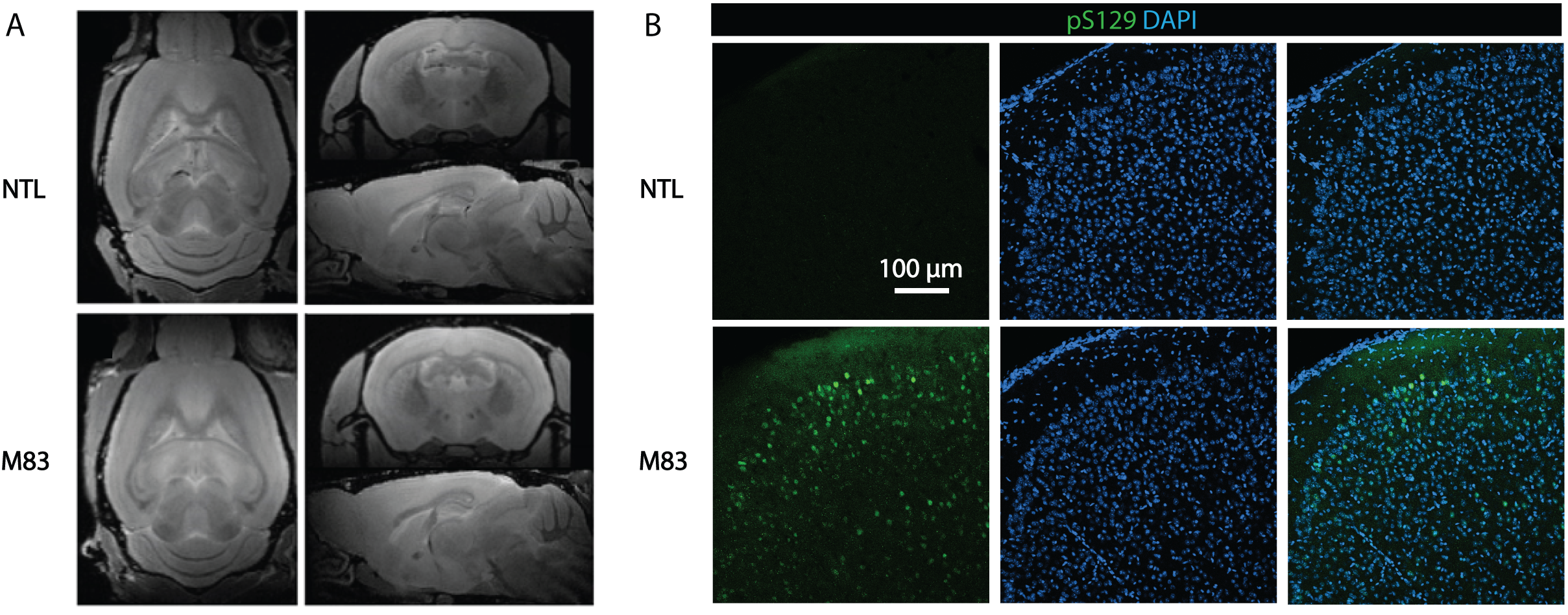

Supplement: Supplementary file 2 — Supplementary Material 2 [file 259_2024_6938_MOESM2_ESM.tif]

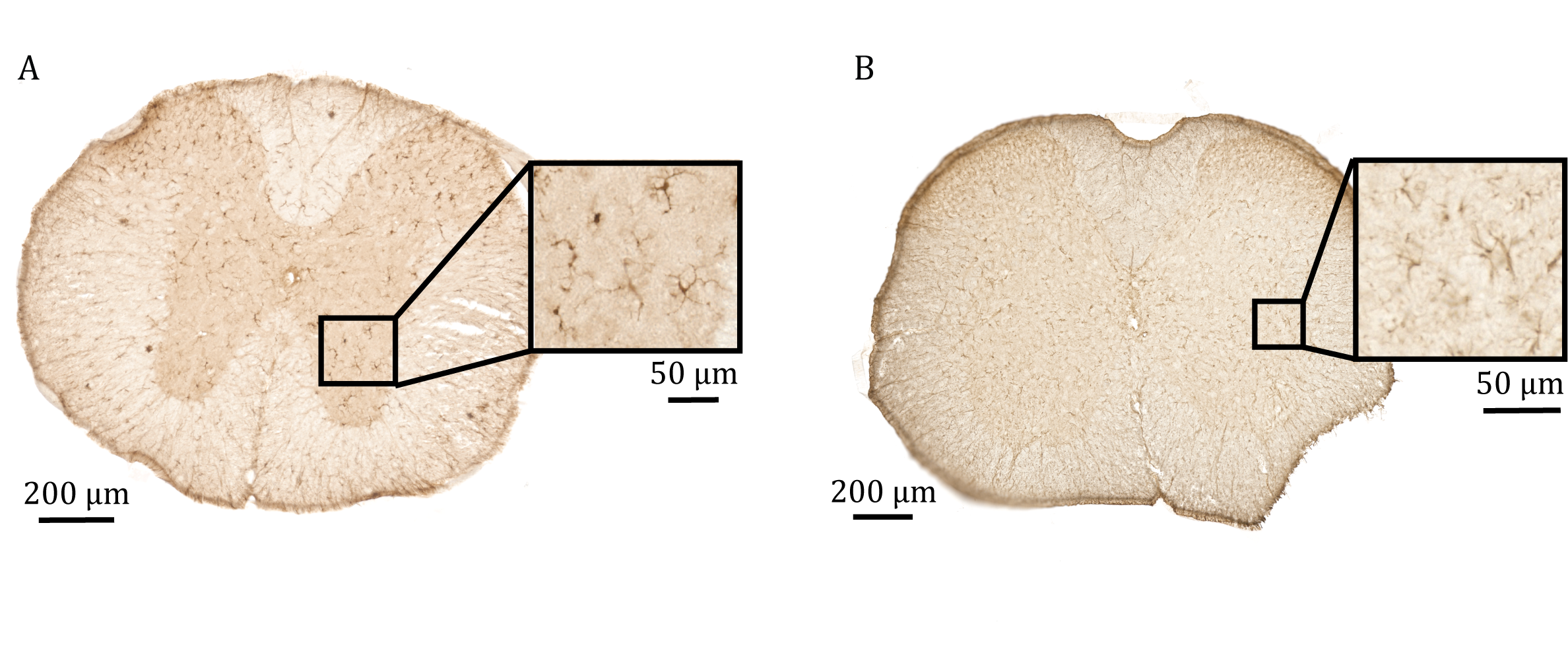

Supplement: Supplementary file 3 — Supplementary Material 3 [file 259_2024_6938_MOESM3_ESM.tif]

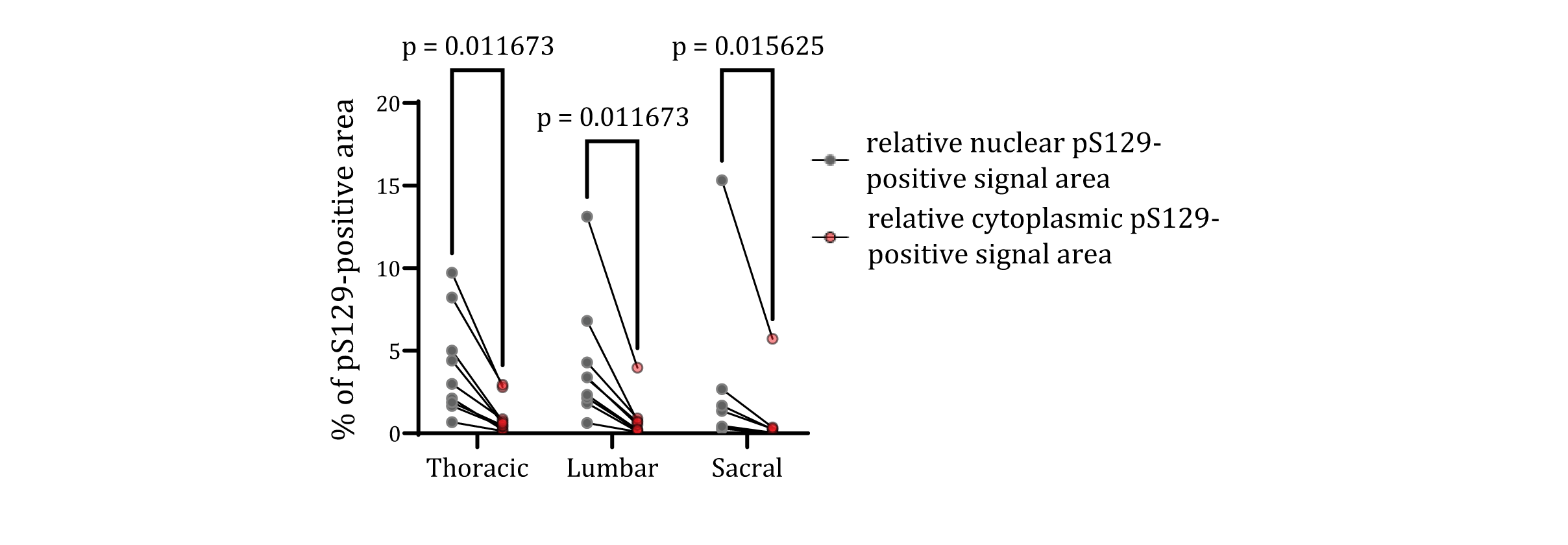

Supplement: Supplementary file 4 — Supplementary Material 4 [file 259_2024_6938_MOESM4_ESM.tif]

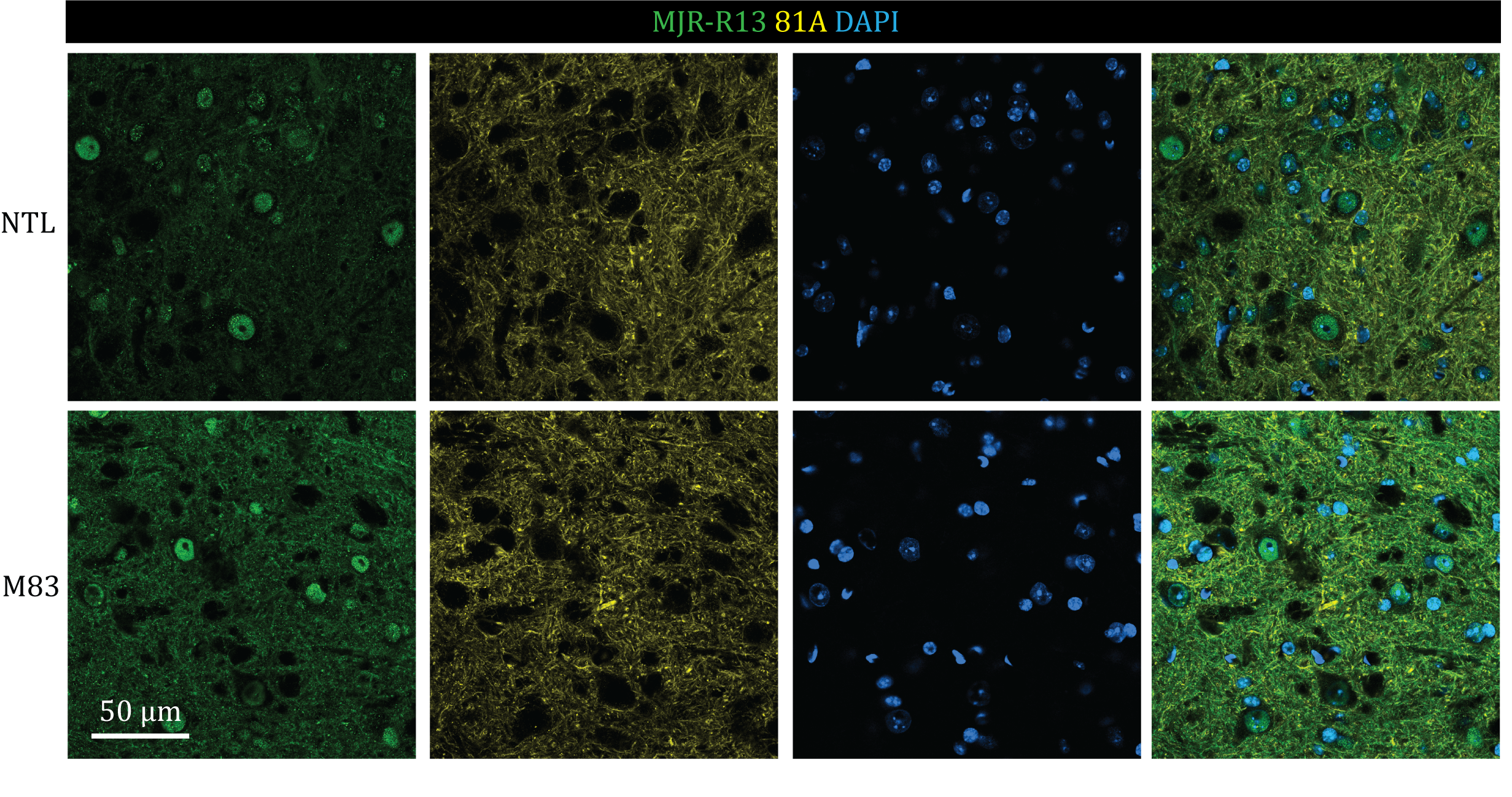

Supplement: Supplementary file 5 — Supplementary Material 5 [file 259_2024_6938_MOESM5_ESM.tif]
