# Supplementary material for: Spiral volumetric optoacoustic tomography of reduced oxygen saturation in the spinal cord of M83 mouse model of Parkinson’s disease
Source: Eur J Nucl Med Mol Imaging. 2024 Oct 9;52(2):427–43. doi: 10.1007/s00259-024-06938-w (PMC11732882; doi:10.1007/s00259-024-06938-w)
Supplement: Supplementary file 6 — Supplementary Material 6 [file 259_2024_6938_MOESM6_ESM.docx]

**Supplementary Table 1. Antibodies, chemicals, and materials used**

| **Reagent** | **Dilution**  **(final concentration)** | **Source** | **Identifier** |
| --- | --- | --- | --- |
| Optimal cutting temperature compound |  | VWR chemicals | 361603E |
| Natural PVA (print core: BB 0.4; layer height: 0.15 mm; print temp: 220_C, bed: 60_C; infill: 20%; build plate adhesion: 3 mm brim) |  | Ultimaker | 1528692-5K |
| NDS |  | Interchim | UP77719A K |
| NGS |  | Jackson ImmunoResearch | 005-000-121 |
| Triton X-100 |  | Merck | RES3103T-A101X |
| Anti-CD31, MEC 13.3 (rat) | 1/50  (0.3125 µg/ml) | BD Pharmingen | 550274  (lot: 1207300) |
| Anti-GLUT1, 5B12.3 (mouse) | 1/1000  (0.5 µg/ml) | Merck Millipore | MABS132  (lot: 3836591) |
| Anti-Iba1 (rabbit) | 1/500  (1-1.4 µg/ml) | Dako | 019-19741  (lot: WTP2670) |
| Anti-alpha-synuclein pS129, 81A (mouse) | 1/1000  (1 µg/ml) | Merck Millipore | MABN826  (lot: 3858247) |
| Anti-alpha-synuclein pS129, MJR-R13 (rabbit) | 1/1000  (4.263 µg/ml) | Abcam | ab168381  (lot: GR3374912-3) |
| Anti-NeuN, A60 (mouse) | 1/100  (10 µg/ml) | Millipore | MAB377  (lot: 2500605) |
| Anti-GFAP (rabbit) | 1/200  (1.7 µg/ml) | Cloud-Clone Corp. | PAA068Mu01  (lot: A20210913001) |
| Anti-alpha-synuclein pS129, EP1536Y (rabbit) | 1/1000  (2.617 µg/ml) | Abcam | ab51253  (lot: GR3378673-20) |
| Antibody anti-mouse – Alexa488 (goat) | 1/500 | Jackson ImmunoResearch | 115-545-003 |
| Antibody anti-rabbit – Alexa488 (donkey) | 1/500 | Jackson ImmunoResearch | 711-545-152 |
| Antibody anti-mouse – Cy3 (donkey) | 1/500 | Jackson ImmunoResearch | 715-165-150 |
| Antibody anti-rat – Cy5 (donkey) | 1/500 | Jackson ImmunoResearch | 712-605-153 |
| Antibody anti-rabbit biotinylated (donkey) | 1/500 | Jackson ImmunoResearch | 711-065-152 |
| ABC-HRP complex |  | Vector Laboratories | PK-6100 |
| DAPI |  | Invitrogen | D1306 |
| ProLong Diamond Antifade Mountant |  | Invitrogen | P36970 |

NDS, normal donkey serum; NGS, normal goat serum; H_2_O_2_, hydrogen peroxide; ABC-HRP, avidin-biodin-horseradish peroxidase; DAPI, 4',6-diamidino-2-phenylindole (DAPI)

**
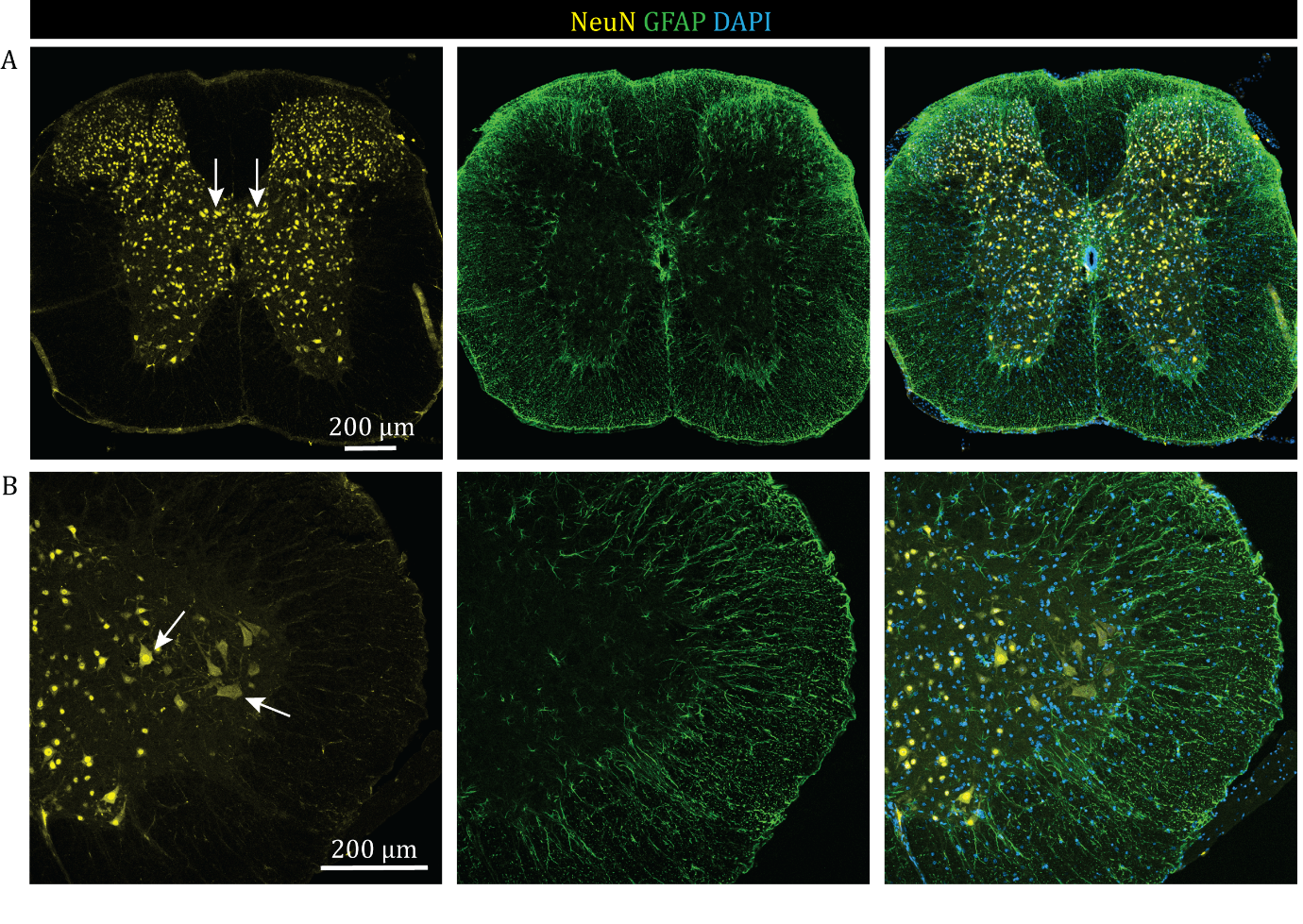
**

**Suppl Fig 1. Neuronal and astroglial staining in the spinal cord of the M83 mouse model:** A) Representative immunofluorescence images of NeuN (yellow) and GFAP (green) in the thoracic spinal region of M83 mice showing intact Clarke’s columns (white arrows) and GFAP-positive astrocytes. B) Representative immunofluorescence images of NeuN and GFAP in the lumbar spinal region of M83 mice showing intact motor neurons (white arrows) and GFAP-positive astrocytes. Nuclei were counterstained with DAPI (blue); scale bars=200 µm.

**
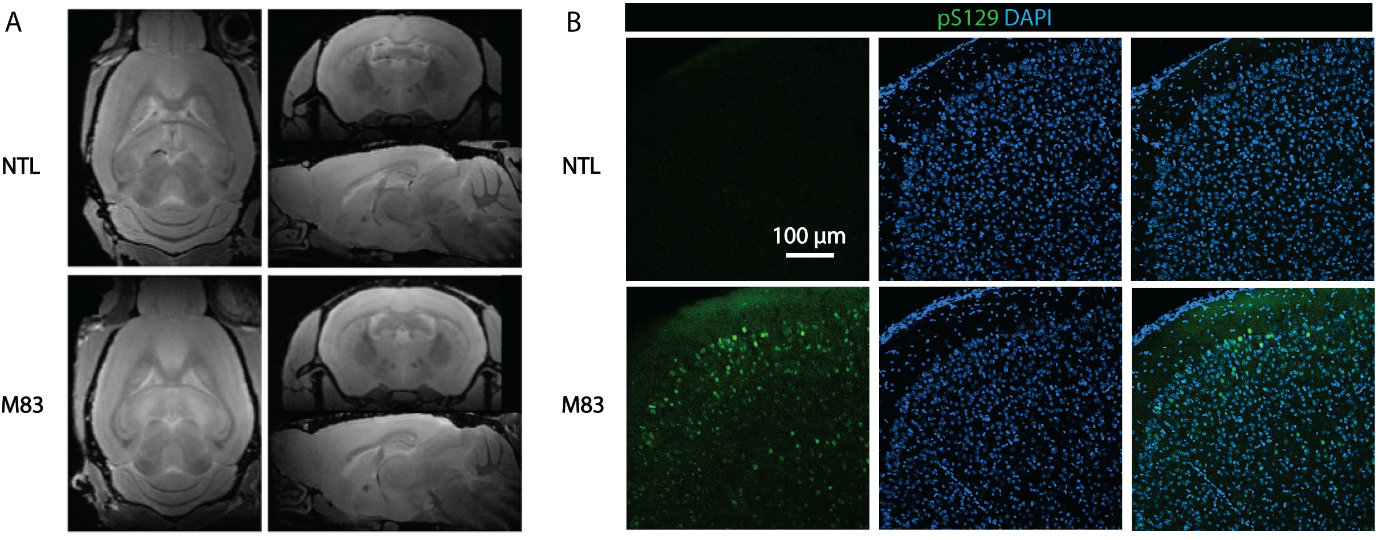
**

**Suppl Fig 2. No regional atrophy in the brains of M83 mice compared to NTL mice, despite the presence of cerebral pS129-positive (clone EP1536Y) α-syn accumulation.** A) High-resolution *ex vivo* T1w MR image acquired at 9.4 T. No apparent atrophy was observed in the brains of M83 mice compared to NTL mice. B) Representative immunofluorescence images of colocalized pS129 (green) and DAPI (blue) showing intranuclear pS129-positive signals in the cortex of the M83 mouse brain, which is absent in the NTL mouse brain; scale bar=100 µm.

**
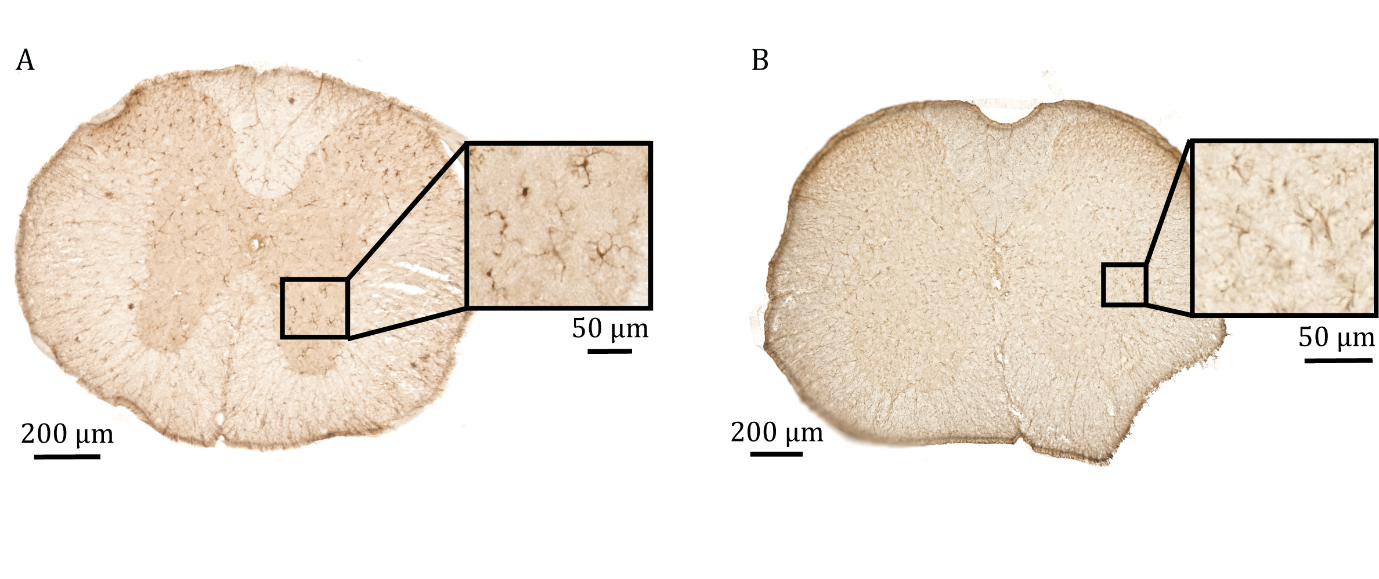
**

**Suppl Fig 3. No apparent microglia or astrocyte activation in the spinal cord of M83 mice:** A) Representative immunohistochemistry images of Iba1-positive microglia in the thoracic spinal segment of the M83 mouse model. B) Representative immunohistochemistry images of GFAP-positive astrocytes in the thoracic spinal segment of the M83 mouse model; scale bars=200 µm and 50 µm.

**
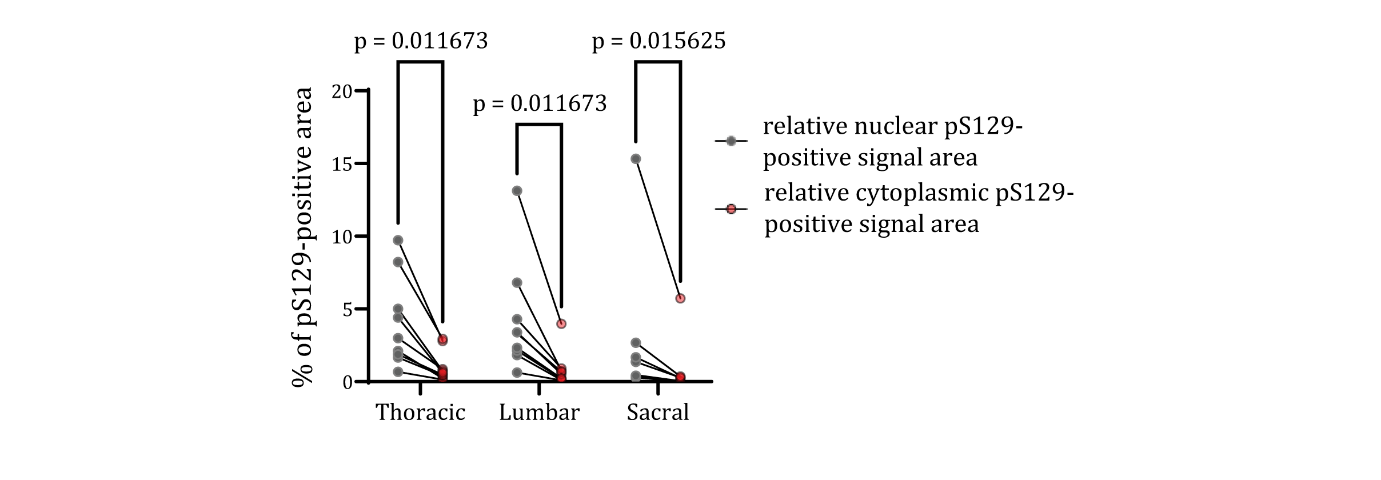
**

**Suppl Fig 4. The pS129-positive signal is located in the vicinity of the nucleus in M83 mice:** There are greater relative nuclear pS129-positive areas (gray) than cytoplasmic pS129-positive areas (red dots) in the M83 thoracic, lumbar and sacral spinal cord segments. N=9 M83. M83: PD mouse model; pS129: α-syn phosphorylated at serine 129.


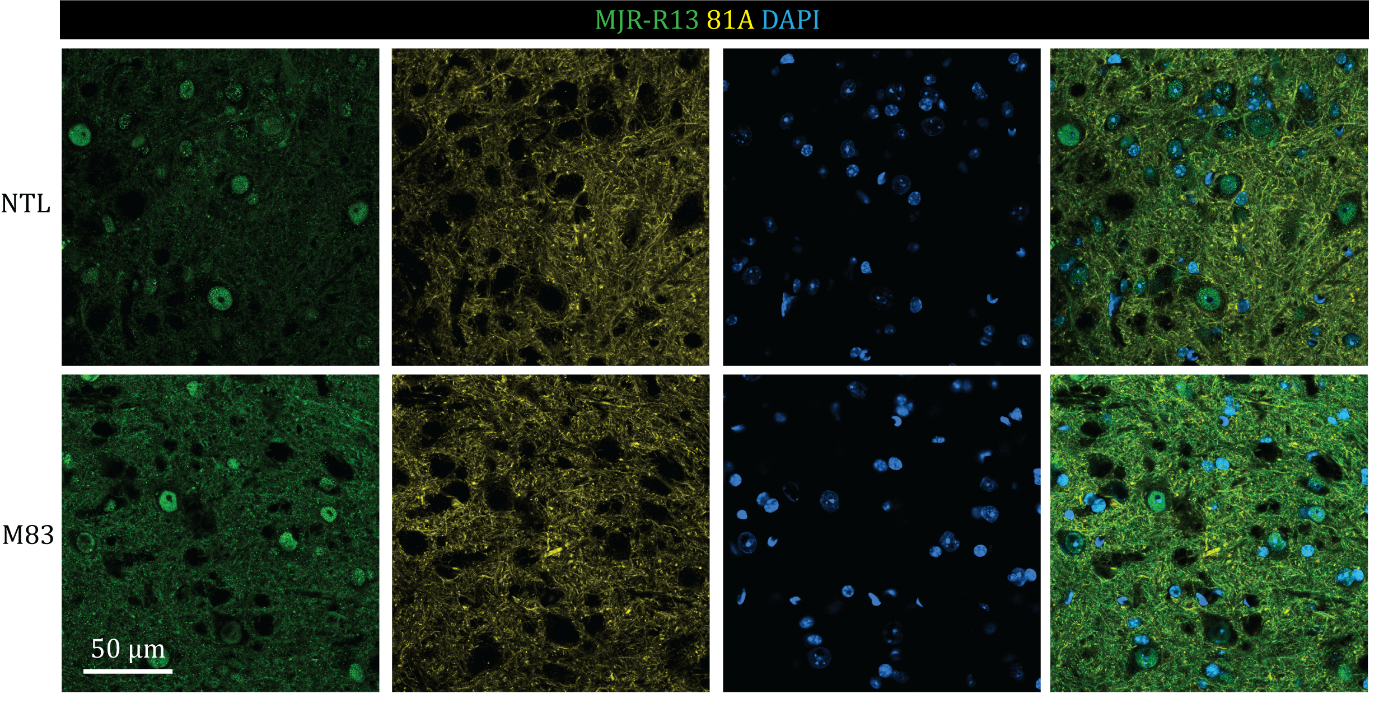


**Suppl Fig 5. No apparent difference in pS129 expression in the spinal cord between M83 mice and NTL mice was revealed by the antibodies against the 81A and MJR-R13 clones.** Representative immunofluorescence images of pS129 targeted with the 81A (yellow) and MJR-R13 (green) clones revealed similar signals in the thoracic spinal cord segment of the M83 mouse model and NTLs. Nuclei were counterstained with DAPI (blue); scale bar=50 µm.
